# Genuine divalent magnesium-ion storage and fast diffusion kinetics in metal oxides at room temperature

**DOI:** 10.1073/pnas.2111549118

**Published:** 2021-09-14

**Authors:** Jinlin Yang, Jibiao Li, Wenbin Gong, Fengxia Geng

**Affiliations:** ^a^College of Energy, Soochow Institute for Energy and Materials Innovations, Soochow University, Suzhou 215006, China;; ^b^College of Materials Science and Engineering, Yangtze Normal University, Chongqing 408100, China;; ^c^School of Physics and Energy, Xuzhou University of Technology, Xuzhou 221018, China

**Keywords:** Mg-ion intercalation chemistry, two-dimensional sheets, metal oxides

## Abstract

Rechargeable magnesium batteries represent a viable alternative to lithium-ion technology that can potentially overcome its safety, cost, and energy density limitations. Nevertheless, the development of a competitive room temperature magnesium battery has been hindered by the sluggish dissociation of electrolyte complexes and the low mobility of Mg^2+^ ions in solids, especially in metal oxides that are generally used in lithium-ion batteries. Herein, we introduce a generic proton-assisted method for the dissociation of the strong Mg–Cl bond to enable genuine Mg^2+^ intercalation into an oxide host lattice; meanwhile, the anisotropic Smoluchowski effect produced by titanium oxide lattices results in unusually fast Mg^2+^ diffusion kinetics along the atomic trough direction with a record high ion conductivity of 1.8 × 10^−4^ S ⋅ cm^−1^ on the same order as polymer electrolyte. The realization of genuine Mg^2+^ storage and fast diffusion kinetics enabled a rare high-power Mg-intercalation battery with inorganic oxides. The success of this work provides important information on engineering surface and interlayer chemistries of layered materials to tackle the sluggish intercalation kinetics of multivalent ions.

## Significance

Rechargeable Mg batteries constitute safe and sustainable high-energy density electrochemical energy storage devices. However, due to an extremely high charge density of Mg^2+^ ions, “real” Mg^2+^-intercalation chemistry has been rarely realized, and significantly decelerated Mg^2+^-diffusion kinetics is always encountered, especially in metal-oxide systems. Herein, we demonstrate a generic strategy to overcome the hitherto insurmountable challenge of developing rechargeable metal-oxide electrode materials for Mg batteries, with genuine Mg^2+^ storage, record fast diffusion kinetics, and excellent cycling performance.

Rechargeable multivalent metal-ion batteries are promising energy sources that can potentially satisfy the existing demand for high-energy density electrochemical energy storage devices (1, 2). The electrochemical discharge and charge reactions in these batteries involve multiple electron transfers per ion, which may significantly increase the ion storage capacity relative to that of monovalent batteries. Among the studied systems, Mg-ion batteries utilizing divalent magnesium ions (Mg^2+^) as charge carriers are considered the most viable option. In addition to their numerous advantages such as abundant Mg resources, low fabrication cost, and environmental friendliness, these batteries exhibit dendrite-free Mg plating and stripping during electrochemical cycling, which ensure high operational safety. Furthermore, the volumetric capacity of Mg anodes (3,833 mAh ⋅ cm^−3^) is almost twice as large as that of Li anodes (2,062 mAh ⋅ cm^−3^) (3–5). Unfortunately, it is very difficult to realize genuine storage and fast transport of Mg^2+^ ions in solids (especially in inorganic oxides) at low temperatures due to their high degree of polarization and charge density. The charge of the Mg^2+^ ion is two times larger than that of the Li^+^ ion, although the ionic radius of Mg^2+^ (0.72 Å) is close to that of Li^+^ (0.76 Å). As a result, Mg^2+^ ions are more likely to form strong covalent bonds with electrolytes (such as Mg–Cl bonds in the commonly used all-phenyl complex electrolytes) with very high dissociation energy. Meanwhile, the strong electrostatic interactions between Mg^2+^ ions and solid host lattices significantly inhibit their diffusion kinetics in these lattices. Resultantly, the migration barrier for Mg^2+^ ions is usually higher than that for Li^+^ ions in the same cathode material (6–8).

To overcome the Mg bond dissociation barrier and enhance Mg-ion diffusion kinetics, intercalation chemistries based on solvated Mg^2+^ as the intercalating cation species, including Mg(DME)_3_^2+^, Mg(H_2_O)_*x*_^2+^, and MgCl^+^, have been established (9–11). These complex ions lower the charge density by either increasing the ionic radius or decreasing the net charge. Although the storage of these complex ions alleviates the drawbacks related to the dissociation and diffusion of bare Mg^2+^ ions, it produces several challenges. The practical energy densities at the cell level for the hybrid battery based on such intercalation chemistry are lower than that of the battery exclusively involving Mg^2+^ storage. Additionally, the coinsertion of these bulky solvent molecules induces significant volume changes of the electrode, thereby limiting its cycle life. Recently, a two-pronged approach has been developed to overcome these challenges (12). It involved the storage of exclusively Mg^2+^ ions and their fast solid-state diffusion in an organic cathode fabricated from pyrene-4,5,9,10-tetraone. Heterogeneous enolization redox chemistry was utilized to avoid the bond cleavage and reformation; meanwhile, an electrolyte comprising weak-coordinated anions in an ethereal solvent blend was employed to increase the bulk ion mobility and promote Mg^2+^ desolvation on the electrode surface. However, it still remains a critical challenge in inorganic materials to overcome the two important problems: the ion dissociation in the conventional Mg chloride complex electrolyte and solid-state ion diffusion. The development of rechargeable inorganic cathodes for Mg batteries using a rational structural design is the major limiting factor of this promising post–Li-ion battery technology.

Metal oxides are the most promising electrode materials for Li-ion batteries, taking advantage of their excellent chemical and thermal stabilities; however, this is not always true for Mg-ion batteries. Compared to sulfides and selenides, most metal oxides suffer from low reversible capacities and slow diffusion kinetics, owing to the higher strength of the Mg–O bond as compared with those of the Mg–S and Mg–Se bonds (7, 13). Furthermore, the reaction of Mg ions with highly polarizable O^2−^ ions often leads to MgO formation rather than Mg^2+^ intercalation, making it almost impossible to achieve reversibility in oxide-based cathodes (14). Therefore, the development of rechargeable metal-oxide electrode materials for Mg-ion batteries characterized by genuine Mg^2+^ storage, fast solid-state diffusion kinetics, and excellent cycling performance (especially at room and low temperatures) remained an unsurmountable challenge.

**Fig. 1.**
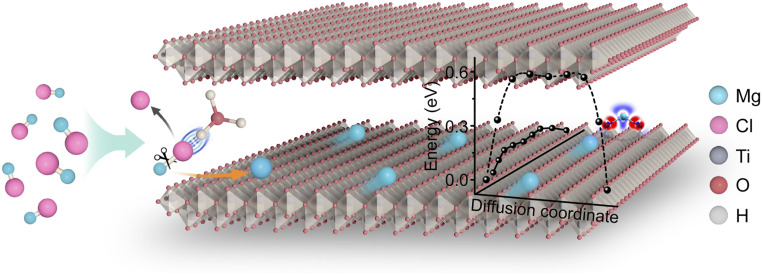
Schematic illustration. The genuine Mg^2+^ storage mechanism and fast diffusion in the oxide electrode, which were studied using PhMgCl–AlCl_3_ electrolyte. The protons efficiently stripped Cl ions from the electrolyte complex, ensuring the genuine Mg^2+^-intercalation chemistry. The anisotropic Smoluchowski effect or wavefunction extension along the atomic troughs on the surface created pathways for the unusually fast diffusion of Mg^2+^ species.

In the present study, genuine Mg^2+^ intercalation/deintercalation and fast diffusion in oxide lattices were realized not only at room temperature but also at subzero temperatures. These outstanding results were achieved by placing protons on negatively charged metal-deficient oxide sheets and disorderly stacking these sheets over a certain distance (this strategy is schematically illustrated in Fig. 1). The stripping of Cl^−^ ions was facilitated by the presence of protons between the sheets, while fast Mg^2+^ diffusion was ensured by the extension of the wavefunction along an atomic trough on the unique sheet surface due to the anisotropic Smoluchowski effect. This induced the formation of flat potential-energy surfaces and diffusion highways, which gave a record high Mg-ion conductivity of 1.8 × 10^−4^ S ⋅ cm^−1^. As a result, the fabricated Mg-ion cell exhibited a high-power density of 7.4 kW ⋅ kg^−1^ while maintaining an energy of 113.0 Wh ⋅ kg^−1^. Practically, the cell battery, which was charged in 55 s, could be gradually discharged for a stable long run of ∼4.5 h. Even at a subzero temperature of −15 °C, the electrode capacity remained above 55%, and the diffusion coefficient was in the range of 10^−9^ tο 10^−11^ cm^2^ ⋅ s^−1^ (10^−8^ to 10^−10^ cm^2^ ⋅ s^−1^ at room temperature). The proposed strategy is generic and can be easily applied to other two-dimensional electrode materials, including titanium oxide, manganese oxide, and oxyanion-terminated titanium carbide.

## Results and Discussion

**Fig. 2.**
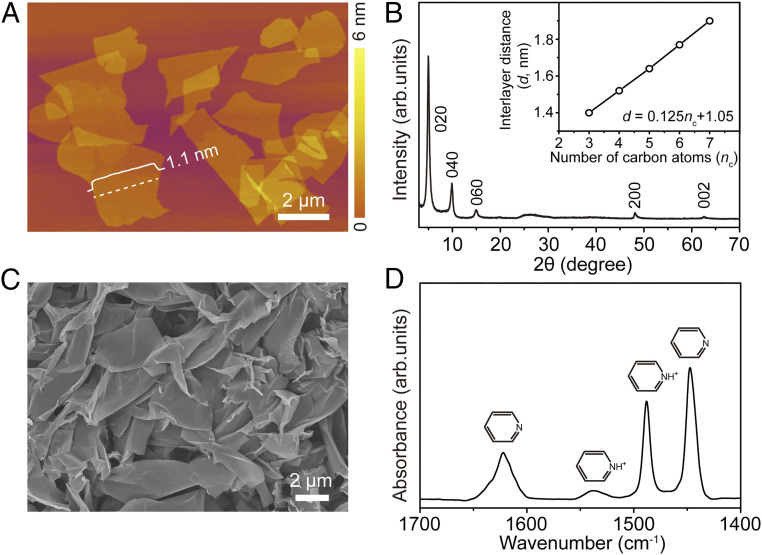
Material structure. (*A*) Representative atomic force microscopy image of the delaminated titanium oxide sheets. (*B*) XRD pattern and (*C*) scanning electron microscopy image of the stacked titanium oxide sheets, which shows a characteristic stacking structure and platelet morphology. (*D*) Fourier-transform infrared spectra of the pyridine molecules adsorbed on the prepared titanium oxide sample at 50 °C. The presence of bands related with pyridinium ions corroborated the high Brønsted acidity of the oxide sample.

Titanium oxide sheets with a net negative charge (Ti_1.74_O_4_^1.04−^) were selected for a representative study. These sheets were obtained via soft chemical delamination using layered K_0.8_Ti_1.73_Li_0.27_O_4_ as a precursor, being protonated in acid and delaminated in tetramethylammonium hydroxide (*SI Appendix*, Fig. S1) (15). A certain number of protons and electrolyte traces remained as residues on the sheet surfaces. The protons could be in the form of hydronium ions or hydroxyl being adsorbed on oxygen atoms at the vertexes of the TiO_6_ octahedra. The atomic force microscopy images revealed that the synthesized titanium oxide sheets had lateral sizes of 2 to 6 µm and thicknesses of 1.1 nm (Fig. 2*A*). The thickness value was consistent with the sum of the crystallographic parameters determined for a single titanium oxide layer (0.75 nm) and the adsorbed ions (16, 17). The Ti_1.74_O_4_^1.04−^ sheets exhibited a high density of net negative charges (9.12 nm^−2^), which enabled electrostatic self-assembly with the positively charged guests such as protonated amines (*SI Appendix*, Fig. S2). A series of primary amines with various alkyl-chain lengths were investigated to determine the states of guest amines in the galleries. The presence of two X-ray diffraction (XRD) peaks corresponding to the (200) and (002) intrasheet reflections indicated that the structure of elementary lepidocrocite sheets was maintained throughout the entire process (Fig. 2*B* and *SI Appendix*, Fig. S3). An analysis on the interlayer gap-dependent (00*l*) stacking reflections along with density functional theory (DFT) calculations revealed that alkyl chains were likely arranged in a bilayer, paraffin-like conformation, which is a typical structure of layered materials (*SI Appendix*, Figs. S3 and S4) (18). The tilting angle was ∼30°. The absence of general (*hkl*) reflections indicated a disordered stacking of the sheets and the absence of a registry between the neighboring sheets. The sheets protonated with the amines also showed step increment in thickness, confirming the interaction of the ammonium ions with the negatively charged titanium oxide sheets (*SI Appendix*, Fig. S5). The hexylammonium-spaced specimen was selected for further detailed studies owing to its optimal electrochemical properties.

The hexylammonium-spaced specimens were described by the formula (Ti_1.74_O_4_)^1.04−^(C_6_H_16_N)^+^_0.36_(H^+^)_0.68_ · 1.37H_2_O (*SI Appendix*, Fig. S6), containing one amine group for every 2.77 unit cells of titanium oxide. The results of scanning electron microscopy observations revealed that the obtained specimen exhibited a platelet-like morphology with a lateral size of 2 to 10 µm and thickness of 10 to 20 nm (Fig. 2*C* and *SI Appendix*, Fig. S7). From the interlayer spacing of 1.77 nm estimated by XRD, it was found that each platelet comprised a stack of 17 sheets. Cross-sectional high-resolution transmission electron microscopy (HR–TEM) images indicated the presence of a stacking structure with an interlayer distance of 1.7 nm (*SI Appendix*, Fig. S8), which was in good agreement with the XRD data. The Fourier-transform infrared spectroscopy (FTIR) and Raman spectra confirmed the hybridization of the titanium oxide phase with protonated alkylamines (*SI Appendix*, Fig. S9). As the dissociation energy of the Mg–Cl bond was significantly lowered in the presence of protons (vide post, see Fig. 5*E* and related explanations), the ability of the oxide structure to generate protons (Brønsted acidity) was evaluated by studying the adsorption of weakly basic pyridine probe, assuming that this process could occur only at highly acidic sites. The dominant IR bands located at 1,488 and 1,540 cm^−1^ in Fig. 2*D* are characteristic of pyridinium ions (protonated pyridine), indicating a high Brønsted acidity, which likely originated from the presence of protons between the layers (19). The obtained results revealed that the analyzed structure comprised a disordered lamellar stack of titanium oxide sheets with tilted alkylamines and protons in the galleries.

**Fig. 3.**
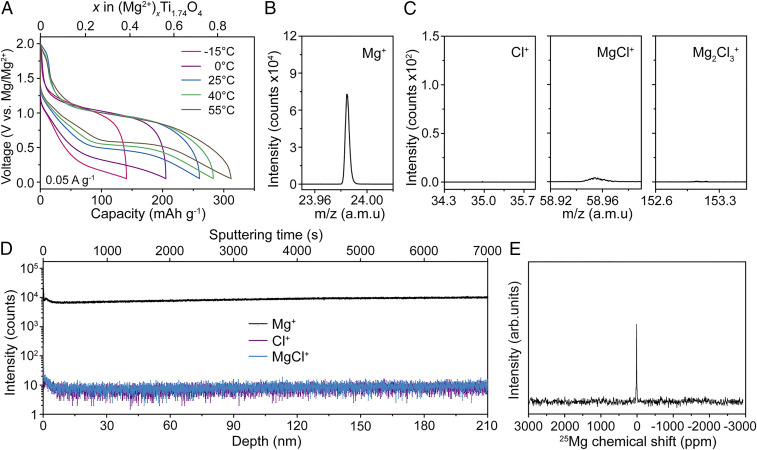
Genuine Mg^2+^ (de)intercalation in the oxide electrode. (*A*) Voltage–capacity profiles obtained for the Mg_*x*_Ti_1.74_O_4_ electrode at temperatures between −15 and 55 °C. The values of *x* were estimated via inductively coupled plasma elemental analysis. TOF–SIMS profiles recorded for the (*B*) positive Mg ions and (*C*) Cl-containing ions emitted from the discharged electrode. The secondary ions (i.e., Mg^+^ and Cl^+^) were generated via the interactions with the Bi^3+^ beam used for analysis. (*D*) TOF–SIMS depth profiles of various chemical species present in the discharged electrode. The specimen depth was calculated based on the calibrated Cs^+^-sputtering rate of ∼0.03 nm ⋅ s^−1^. (*E*) ^25^Mg NMR spectrum of the discharged electrode.

The electrochemical activity of the designed oxide electrode for Mg-ion storage was evaluated using a coin cell with an Mg anode. A standard all-phenyl Mg chloride complex electrolyte (0.4 M 2PhMgCl–AlCl_3_ in tetrahydrofuran) was utilized due to its high Coulombic efficiency for the reversible deposition of Mg (*SI Appendix*, Fig. S10). The estimated theoretical capacity based on complete reduction of Ti^4+^ to Ti^3+^ was ∼317 mAh ⋅ g^−1^ (1.73 charges per formula unit). Fig. 3*A* displays the charge–discharge profiles recorded at a low current rate of 0.05 A ⋅ g^−1^ after an activation cycle (*SI Appendix*, Fig. S11). Although divalent Mg ions generally exhibit low mobility, and a relatively high temperature is required to achieve a satisfactory battery capacity, a discharge capacity of 260 mAh ⋅ g^−1^ was obtained at a practical current density of 50 mA ⋅ g^−1^ (0.16 C) and room temperature (25 °C). The obtained discharge capacity corresponded to 1.42 electron transfers per formula unit (82% of the theoretical value). The results of inductively coupled plasma–atomic emission spectroscopy measurements revealed that the Mg content in the studied sample was 0.76 (*SI Appendix*, Fig. S12). The compositions of intercalated species were then determined from the charge and Mg content; surprisingly, the main intercalated included divalent Mg^2+^ and MgCl^+^ ions with a molar ratio of 0.66:0.10. A quantitative analysis by energy-dispersive X-ray spectroscopy produced consistent results with a Cl-to-Mg atomic ratio of 0.12 (*SI Appendix*, Fig. S12). Time-of-flight secondary ion mass spectrometry (TOF–SIMS) is a powerful technique for the identification of elements at the atomic level, with a high sensitivity corresponding to a detection limit in the parts per billion range (20). In the obtained TOF–SIMS profiles, the signal generated by Mg^+^ ions was detected at *m/z* = 24. However, the amounts of Cl^−^ or electrolyte complex ions, including MgCl^+^ and Mg_2_Cl_3_^+^, were negligible, which confirmed that Mg^2+^ ions were the primary active species (Fig. 3 *B* and *C*). All the detected species were distributed homogenously throughout the electrode bulk, as shown in the TOF–SIMS sputter depth profiles of the 210-nm-thick film depicted in Fig. 3*D*. The obtained ^25^Mg magic-angle spinning NMR spectra also exhibit no detectable signals due to Mg–Cl species (Fig. 3*E*). Thus, MgCl^+^ ions were not among the active cations in the studied electrochemical reactions. In addition, no signal corresponding to MgO species (at ∼26 ppm) was detected, which precluded the formation of hexa-coordinated MgO species (21). Therefore, the present study demonstrates a genuine divalent Mg-ion intercalation into an inorganic oxide lattice.

Notably, the subsequent charging process induced the deintercalation of almost the entire Mg amount with a Coulombic efficiency of ∼100%. The estimated ratios between the Ti_1.74_O_4_, Mg, C_6_H_16_N, and H_2_O components in the fully discharged and charged electrodes were equal to 1:0.76:0.09:1.1 and 1:0.07:0.09:1.1, respectively (*SI Appendix*, Fig. S13). The content of hexylammonium cations remained unchanged, which helped maintain the stacking structure. Furthermore, the lattice water was retained in the lattice without penetrating the electrolyte. The effect of temperature on the cell performance was investigated as well. At an operating temperature of 55 °C, the cell exhibited a high capacity of 312 mAh ⋅ g^−1^ (1.7 charges per formula unit), which was close to the theoretical value. More impressively, high-capacity retention was observed at low operating temperatures (Fig. 3*A*), revealing that 55% of the cell capacity at a room temperature of 25 °C was retained at a subzero temperature of −15 °C. The capacity retention properties of the fabricated electrode at a subzero temperature were superior to those reported previously for TiS_2_ (28%) (11) and VS_4_ (15%) (22) electrodes.

**Fig. 4.**
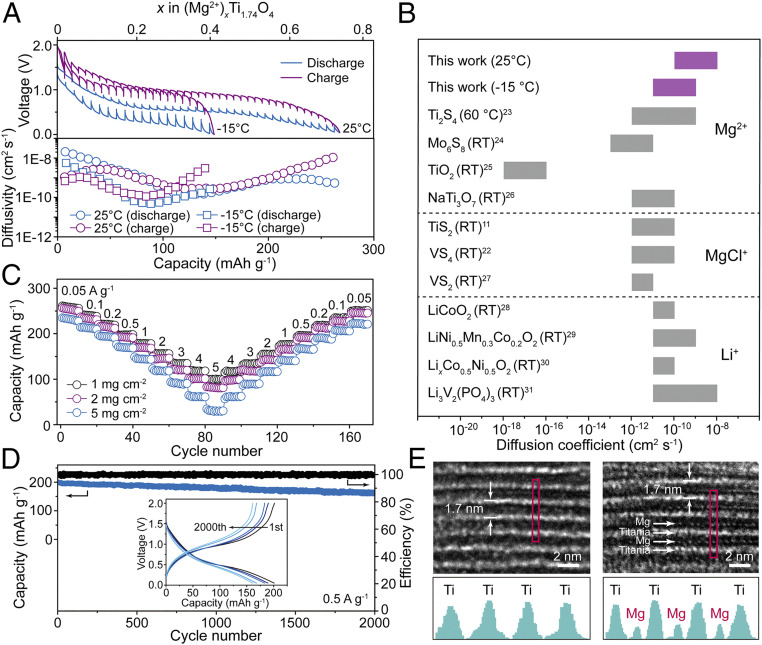
Fast and reversible diffusion of Mg^2+^ in the oxide electrode. (*A*) Galvanostatic intermittent titration technique curves and calculated Mg^2+^ diffusion coefficients plotted as functions of the electrode stoichiometry at temperatures of 25 and −15 °C. (*B*) Diffusion coefficients of the electrode material developed in the present study, electrode materials for representative Mg batteries (including both divalent Mg-ion and monovalent MgCl-ion batteries), and electrode materials for Li batteries. (*C*) Rate capabilities measured at charge/discharge rates of 50 to 5,000 mA ⋅ g^−1^ (16 C) and mass loadings of 1, 2, and 5 mg ⋅ cm^−2^. (*D*) Electrode cycling performance at a charge/discharge rate of 500 mA ⋅ g^−1^. The electrode was activated for three cycles at 50 mA ⋅ g^−1^ before the cycling test. (*Inset*) Voltage–capacity profiles obtained during cycling. (*E*) Representative cross-sectional HR–TEM images and intensity profiles of the titanium oxide electrode obtained before and after full magnesiation.

The high charge density of divalent Mg cations generally results in slow solid-state diffusion (especially in metal-oxide lattices), which significantly lowers the power output of Mg batteries. The diffusion coefficient of the system developed in the present study was estimated using a galvanostatic intermittent titration technique. The Mg^2+^ diffusivity decreased from 2.1 × 10^−8^ to 1.5 × 10^−10^ cm^2^ ⋅ s^−1^, with an increase in the Mg^2+^ concentration in the host lattice (Fig. 4*A*). This decrease was likely caused by electrostatic repulsion; however, the observed range of the Mg^2+^ diffusion coefficient indicated relatively fast charge transfer kinetics. Its values obtained for the designed experimental system were ∼10^2^ to 10^3^ higher than those of previously reported Mg^2+^-intercalation materials (23–26), including cubic thiospinel Ti_2_S_4_ and Chevrel phase Mo_6_S_8_ (∼10^−10^ to 10^−13^ cm^2^ ⋅ s^−1^) and even comparable with MgCl^+^ and Li^+^ diffusivity (∼10^−10^ cm^2^ ⋅ s^−1^) (11, 22, 27–31), as summarized in Fig. 4*B*. Strikingly, as the temperature dropped to −15 °C, the Mg^2+^ diffusion coefficient remained in the range of 10^−9^ to 10^−11^ cm^2^ ⋅ s^−1^, validating the rapid ion diffusion in the studied system. The ion conductivity was also examined with typical impedance spectroscopy measurements, which gave a value of 1.8 × 10^−4^ S ⋅ cm^−1^ at room temperature (*SI Appendix*, Fig. S14), although a high temperature of >500 °C is typically essential for enabling Mg^2+^ transportation. The contributions from both electronic conductivity and motion of protons were negligible (*SI Appendix*, Fig. S15). Comparing with all reports as far as we know, this is still the highest record, even magnitudes higher than excellent ion-conducting metal−organic framework materials (*SI Appendix*, Table S1).

The fast diffusion kinetics in the prepared titanium oxide electrode resulted in stable and high-rate capability, even in the case of thick electrodes with mass loadings ranging from 1 to 5 mg ⋅ cm^−2^ (Fig. 4*C*). At a typical loading amount used in research studies (1 mg ⋅ cm^−2^), the electrode exhibited good capacity retention varying from 260 to 241, 221, 198, 178, 156, 135, 118, and 100 mAh ⋅ g^−1^ as the current density progressively increased from 50 to 100, 200, 500, 1,000, 2,000, 3,000, 4,000, and 5,000 mA ⋅ g^−1^ (16 C), respectively. Even at a high mass loading of 5 mg ⋅ cm^−2^, the discharge capacity reached a high value of 240 mAh ⋅ g^−1^ at a current density of 50 mA ⋅ g^−1^ operated in lean electrolyte conditions (7.6 μL ⋅ mg^−1^), illustrating the feasibility of fabricating high-mass loading electrodes with high energy and power performance. The obtained maximum power density was 7.4 kW ⋅ kg^−1^ while maintaining an energy of 113.0 Wh ⋅ kg^−1^, which was the highest value ever reported for inorganic Mg^2+^ and MgCl^+^ storage systems (*SI Appendix*, Table S2). Practically, the cell that was rapidly charged at 15 A ⋅ g^−1^ (47 C) in 55 s could stably run for over 4.5 h at a current of 0.05 A ⋅ g^−1^ (0.16 C) (*SI Appendix*, Fig. S16). This is quite appealing for many real-world applications, such as smartphones obtaining an adequate battery life by recharge within 1 min.

The electronegative O atoms in the host lattices of typical oxides tend to trap Mg^2+^ ions, which prevents their release during charging reactions, resulting in the occurrence of irreversible reactions and poor cycling performance (6, 32). Impressively, the oxide electrode developed in this study demonstrated excellent long-term stability. At a current density of 500 mA ⋅ g^−1^ (1.6 C) and extended run over 2,000 cycles, the capacity retention was 81% with a decay rate of 0.0095% per cycle (Fig. 4*D*). When the operating temperature decreased to −15 °C, the electrode exhibited a capacity decay rate of 0.05% per cycle with a capacity of 113 mAh ⋅ g^−1^ after 400 cycles (*SI Appendix*, Fig. S17). Consistent with this performance, the shapes of the discharge/charge profiles were preserved after long-term cycling, and the Mg electrode retained a dendrite-free surface morphology (see Fig. 4*D*, *Inset* and *SI Appendix*, Fig. S18). To the best of our knowledge, the rate capability and cycling stability of the electrode material fabricated in this study were superior to those of all the previously reported inorganic electrode materials for Mg-ion batteries (*SI Appendix*, Fig. S19 and Table S3). In situ XRD analysis showed that neither phase transformation nor significant volume change occurred during a typical charge/discharge cycle (*SI Appendix*, Fig. S20), suggesting a zero-strain insertion/extraction of ions. The cross-sectional HR–TEM image of the fully discharged electrode also revealed that the layered structure with a spacing of 1.7 nm was well maintained, although Mg ions were apparently intercalated between the layers (Fig. 4*E*). Such a quasi–zero-strain characteristic resulted from the pillaring effect of the interlayer alkyl–ammonium ions, which in turn accounted for the excellent long-term cycling stability of the oxide electrode.

**Fig. 5.**
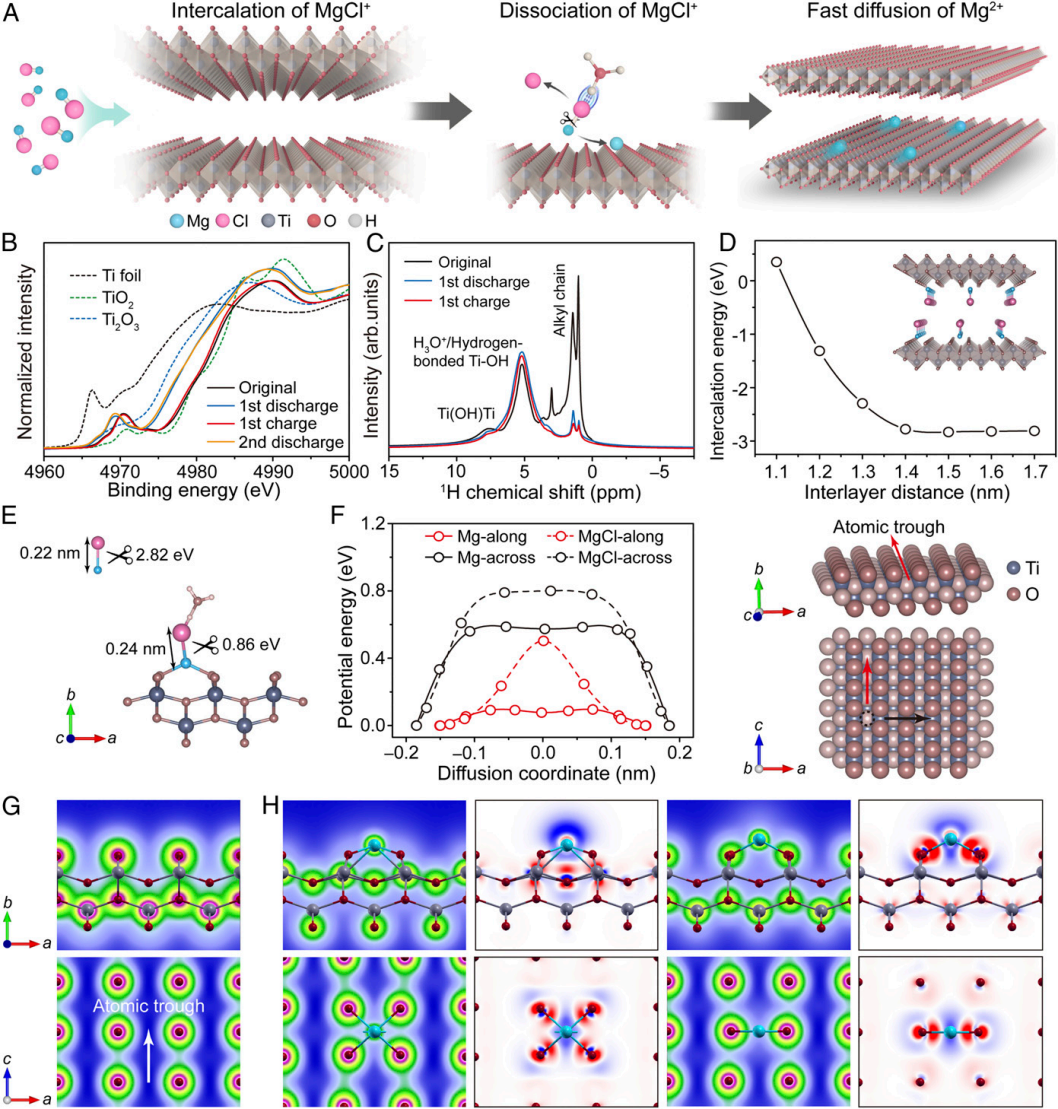
Mechanism for genuine Mg^2+^ intercalation and fast diffusion. (*A*) Schematic illustration of the mechanism of Mg^2+^ storage and fast diffusion in the designed oxide electrode evaluated using PhMgCl–AlCl_3_ electrolyte. (*B*) XANES spectra of the designed titanium oxide electrode recorded at the Ti K-edge in different charged and discharged states. (*C*) ^1^H NMR spectra of the pristine, discharged, and charged electrodes. (*D*) Intercalation barrier of MgCl^+^ ions plotted as a function of the interlayer distance. (*E*) Desolvation energy of MgCl species and the related change in their bond length. (*F*) Potential-energy surfaces obtained for Mg^2+^ and MgCl^+^ ions along different diffusion paths. (*G*) Electronic density profiles of the titanium oxide structure. (*H*) Electronic density profiles of the titanium oxide structure with the added Mg atoms and the corresponding differences in the charge density. Red: charge accumulation, blue: charge depletion.

The primary oxidation state of the pristine electrode was Ti^4+^, while the fully discharged electrode exhibited a shift to low energies and reduction to a valence state near Ti^3+^. This change in the Ti valence state was reversible during the alternate magnesiation/demagnesiation processes (Fig. 5*B*). The participation of oxygen atoms, solvent tetrahydrofuran molecules, and protons were all excluded (Fig. 5*C* and *SI Appendix*, Figs. S21 and S22). In the first discharging, a partial extraction of alkylammonium ions occurred, which produced vacant sites for Mg-intercalation. The results of DFT calculations revealed that the residual amount of C_6_H_16_N:Ti_1.74_O_4_ (=0.09:1) was sufficient to stabilize the stacking structure with a high interlayer distance of 1.7 nm (*SI Appendix*, Fig. S23). The amines remained almost intact during the subsequent discharge and charge cycles, and there were no variations in the corresponding signal intensities and chemical compositions, which may help stabilize the structure. Furthermore, the intensities of the ^1^H NMR signals generated by hydroxyl groups and the lattice water remained essentially the same during electrochemical cycling, demonstrating high stability of the lattice water in the electrode structure and also excluding the involvement of protons during the reversible charge and discharge reactions.

Based on these results, it was concluded that the ion storage process likely involved three steps: MgCl^+^ intercalation, Mg–Cl dissociation into Mg^2+^ and Cl^−^ ions, and fast Mg^2+^ diffusion in the titanium oxide lattice (Fig. 5*A*). In almost all Cl-based electrolyte systems, Mg ions typically exist in a complex form (MgCl^+^ or minor Mg_2_Cl_3_^+^), the dissociation of which requires a high energy of ∼3.0 eV. Thus, various MgCl_*x*_^+^ clusters, rather than Mg^2+^ ions, were confirmed to be the active cations participating in the electrochemical reactions when the host lattice possessed a sufficient space to accommodate bulky ions (11, 33, 34). The intercalation barrier for the bulky MgCl^+^ ions with an ionic radius of 0.226 nm in the developed electrode material was estimated through DFT calculations. A substantial decrease to −2.7 eV was observed when the interlayer distance exceeded 1.4 nm (Fig. 5*D*), which roughly corresponds to the accommodation of two layers of MgCl. Experimentally, the amount of Mg ions that could be intercalated into the gallery (intercalation capacity) was strongly dependent on the interlayer distance. For the samples with larger interlayer distances, the amount of intercalated Mg significantly increased under the same measurement conditions (*SI Appendix*, Figs. S24 and S25). The effect of specific surface was excluded from consideration because all the alkylammonium-spaced specimens possessed similar specific surface areas (*SI Appendix*, Fig. S26). These results suggest that the expanded interlayer facilitates the direct intercalation of MgCl^+^ ions during the first step. Subsequently, the compositions of intercalated species in all structures after the full discharge were determined. Regardless of the amine length, divalent Mg^2+^ ions were mainly observed (*SI Appendix*, Fig. S27), which suggested that the stripping of Cl ions was likely related to the oxide host, probably protons on the oxide sheets. The calculated cleavage energy of Mg–Cl bonds in the presence of H_3_O^+^ ions decreased significantly from 2.82 to 0.86 eV, and the bond distance increased by 9% from 0.22 to 0.24 nm (Fig. 5*E*), indicating a high probability of Mg–Cl dissociation in the presence of protons.

To elucidate the third step of the unusually fast diffusion of divalent Mg^2+^ ions in oxide lattices, the diffusion energy barrier was calculated (Fig. 5*F* and *SI Appendix*, Fig. S28). Because titanium oxide sheets were stacked in a disordered manner over large distances, the transport behavior of Mg^2+^ ions across a titanium oxide sheet was considered. It was found that these ions preferred atomic troughs in the lattice as diffusion pathways with an energy barrier below 0.1 eV. Moreover, the top region of the minimum energy path for the Mg^2+^ transport along this direction was essentially flat. Across the atomic troughs, the diffusion barriers of Mg^2+^ ions significantly increased to 0.6 eV, although the top region of the minimum energy paths remained flat. Unfortunately, for the diffusion of MgCl^+^ ions with a lower charge density, this surface did not provide any benefits. The MgCl^+^ diffusion barrier was higher than that for Mg^2+^ ions along the atomic troughs, and a high singlet barrier of 0.5 eV was detected for the former species, inhibiting its fast migration. By a comparison with reported materials, it was noted that the energy barrier for the Mg^2+^ migration across the titanium oxide sheet in this study was lower than that of the Mg^2+^ diffusion across TiS_2_ (11), MoS_2_ (35), and even Mo_6_S_8_ (36) (the latter represents a well-known material with anomalously fast Mg^2+^ transport). The electronic origins of fast Mg^2+^ diffusion were investigated via density analysis, and the obtained results are presented in Fig. 5 *G* and *H*. The sp electrons in the analyzed titanium oxides were preferentially “spilled-out” along the atomic troughs owing to the anisotropic Smoluchowski effect. This effect became more pronounced with the addition of Mg^2+^ ions at both the hollow and bridge sites along the troughs. The extension of the wavefunction induced the formation of flat potential-energy surfaces along the atomic troughs, thereby providing a solid foundation for the unusually fast Mg^2+^ diffusion in this direction. This also fundamentally explains the absence of MgO formation during Mg^2+^ intercalation and diffusion. Electrochemical impedance spectra indicate a significant decrease in the radius of the semicircle in the high-medium frequency region due to Mg^2+^ intercalation, implying a small charge transfer resistance and high probability of achieving fast reaction kinetics (*SI Appendix*, Fig. S29). The spill-out of electrons changed the electron distribution and surface polarizability, which in turn caused a slight structural distortion in the Ti coordination of Ti–O and Ti–Ti bonds (*SI Appendix*, Fig. S30). The lattice water and/or hydroxyl groups might also help screen Coulombic charges, and the dehydrated sample possessed lower discharge and charge capacities and rate capabilities (*SI Appendix*, Fig. S31). Hence, the unique electrode surface and low diffusion energy barrier account for the fast diffusion of bare Mg^2+^ ions at room and even subzero temperatures.

Intriguingly, the proton-assisted technique utilized to achieve genuine divalent Mg-ion storage can be generally applied to other two-dimensional materials, including manganese oxide and oxyanion-terminated titanium carbide (*SI Appendix*, Figs. S32–S35). In these cases, the predominant intercalating species switched from MgCl^+^ to Mg^2+^ with the incorporation of protons into the gallery. In addition, the diffusivity of divalent Mg^2+^ ions was comparable to that of monovalent MgCl^+^ ions, exhibiting no apparent degradation in the migration rate.

In summary, both genuine intercalation and fast diffusion of divalent Mg ions were achieved in metal oxides not only at room temperature but also at a subzero temperature in the present study. The proposed strategy involved placing protons on metal-oxide sheets and stacking these sheets over a large distance in a disordered manner. The high distance promoted the intercalation of Mg complexes; the protons triggered the cleavage of the Mg–Cl bond; and the anisotropic Smoluchowski effect (the wavefunction extension in the atomic trough of the titanium oxide lattice) produced flat potential-energy surfaces, which enabled unusually fast diffusion of divalent Mg^2+^ species with a record Mg-ion conductivity of 1.8 × 10^−4^ S ⋅ cm^−1^ at a room temperature of 25 °C. As a result, a high-power Mg-ion battery that maintains the high-energy profit was obtained. Practically, the cell that was charged in 55 s could be gradually discharged for a stable long run of ∼4.5 h. Importantly, the demonstrated approach can be extended to other two-dimensional sheet materials, representing a general strategy for designing high-performance electrodes for multivalent metal-ion batteries with superior diffusion characteristics.

## Materials and Methods

Experimental details on the procedures of preparing titanium oxide sheets and *n*-alkylammonium ion-spaced oxide specimens, structural characterization, collecting electrochemical performance data, calculating the diffusion coefficient, estimating energy and power densities, and the settings for theoretical simulations are provided in *SI Appendix*.

## Supplementary Material

Supplementary File

## Data Availability

All study data are included in the article and/or *SI Appendix*.
