# Supplementary material for: Genuine divalent magnesium-ion storage and fast diffusion kinetics in metal oxides at room temperature
Source: Proc Natl Acad Sci U S A. 2021 Sep 14;118(38):e2111549118. doi: 10.1073/pnas.2111549118 (PMC8463793; doi:10.1073/pnas.2111549118)
Supplement: Supplementary File [file pnas.2111549118.sapp.pdf]

PNAS
www.pnas.org

Supplementary Information for

Genuine Divalent Magnesium-Ion Storage and Fast Diffusion Kinetics in Metal Oxides at Room Temperature

Jinlin Yang, Jibiao Li, Wenbin Gong, Fengxia Geng*
Email: gengfx@suda.edu.cn

This PDF file includes:

Experimental section
Figures S1 to S35
Tables S1 to S3
SI References

EXPERIMENTAL SECTION

Preparation of titanium oxide sheets

Titanium oxide ($Ti_{1.74}O_4^{1.04-}$) nanosheets were synthesized via the delamination of a layered titanate, $K_{0.8}Ti_{1.73}Li_{0.27}O_4$, using a soft chemical procedure. A mixture of TiO_2 (Sigma-Aldrich, ≥ 99.9%), K_2CO_3 (Sigma-Aldrich, ≥ 99.9%), and Li_2CO_3 (Sigma-Aldrich, ≥ 99.9%) powders with a molar ratio of 10.4:2.4:0.8 was loaded into a Pt crucible and then annealed at 800 °C in air for 20 h to obtain a pure $K_{0.8}Ti_{1.73}Li_{0.27}O_4$ phase. The resulting specimen was converted into a protonic form $H_{1.07}Ti_{1.73}O_4$ via the proton exchange with a 0.5 mol L^{-1} HCl solution for 72 h at a room temperature of 25 °C. This procedure was repeated thrice by replacing the acid solution every 24 h. The product was collected via filtration, washed with substantial amounts of water and ethanol, and air-dried. The protonated phase was dispersed in a tetramethylammonium hydroxide ($(CH_3)_4NOH$; TMAOH) solution (Aladdin, ≥ 25 wt%) and shaken at 140 rpm for 7 d at ambient temperature to produce a single-layer titanium oxide dispersion.

Preparation of *n*-alkylammonium ion-spaced oxide specimens

n-alkylammonium ion-spaced titanium oxide specimens were prepared by mixing the aqueous dispersion of titanium oxide nanosheets with aqueous solutions of *n*-alkylamine (Aladdin, ≥ 99%), propylamine, butylamine, pentylamine, hexylamine, and heptylamine. The utilized *n*-alkylamines were protonated into the corresponding ammonium ions and then electrostatically coagulated the negatively charged titanium oxide sheets. The dispersion of the titanium oxide nanosheets (40 mL, 5 mg mL^{-1}) was added dropwise to the alkylamine solution (40 mL, 0.2 mL of liquid *n*-alkylamine) under constant stirring at a room temperature of 25 °C, resulting in immediate flocculation. After stirring the mixture overnight, the precipitate was collected via repeated centrifugation and washing with pure H_2O followed by freeze drying to yield the desired powder.

Characterization

The synthesized specimens were analyzed by powder X-ray diffraction (XRD; D8 Advance, Bruker AXS GmbH, Karlsruhe, Germany) using Cu Kα radiation. Their morphological characterization and elemental composition analyses were performed by scanning electron microscopy (SEM) combined with energy-dispersive X-ray

spectroscopy (EDS; S-4700, FEI Company (Thermo Fisher Scientific), Oregon, USA). Additional elemental analysis was conducted by inductively coupled plasma atomic emission spectroscopy (ICP–AES, Thermo Scientific iCAP 7000), and C, H, and N elements were quantified by an elemental analyzer (Vario MACRO cube, Germany). Thermogravimetric (TG) analysis was conducted on a TG 209 F1 Libra instrument to determine the water contents in the prepared specimens. Measurements were performed in air from the room temperature of 25 °C to 800 °C at a heating rate of 10 °C min^{-1}. The lateral size and thickness of the delaminated titania/MnO_2/Ti_3C_2 nanosheets were determined by tapping mode atomic force microscopy (AFM; Dimension Icon, Bruker Instruments Inc., Darmstadt, Germany). The atomic arrangements in the analyzed specimens were investigated by spherical aberration-corrected high-resolution transmission electron microscopy (Cs-corrected HR–TEM; Titan Themis Cubed G2 300, FEI Company (Thermo Fisher Scientific), Oregon, USA) at 80 kV. The powder specimens were pressed into disks using KBr and then analyzed by Fourier transform infrared (FT–IR) spectroscopy (Tensor 27, Bruker Optik GmbH, Ettlingen, Germany) in the transmission mode. To facilitate pyridine adsorption, the disks were initially pretreated under vacuum at 150 °C for 1 h to eliminate surface contaminants. Thereafter, they were cooled to a room temperature of 25 °C and then exposed to pyridine vapor for 30 min. The FT–IR spectrum of the adsorbed pyridine was collected after desorption at 50 °C under vacuum to determine its Brønsted acidity. Raman spectra were recorded on a Raman spectrometer (HR-800, HORIBA JobinYvon) at an excitation wavelength of 633 nm. In addition, X-ray photoelectron spectroscopy (XPS; Escalab 250Xi, Thermo Fisher Scientific, Massachusetts, USA) was performed using monochromatic Al Kα radiation. Brunauer–Emmett–Teller (BET) specific surface areas were determined from the corresponding N_2 adsorption–desorption isotherms (ASAP 2020, Micromeritics Instrument Corporation, Virginia, USA) recorded at −196 °C. Time-of-flight secondary-ion mass spectrometry (TOF–SIMS) measurements were conducted on a TOF–SIMS 5 instrument (IONTOF GmbH, Münster, Germany). The analysis chamber was maintained under ultra-high vacuum ($< 2 \times 10^{-9}$ mbar). A Bi^{3+} beam (30 keV, 1 pA) was utilized as the primary ion source for surface analysis, and the analyzed area had a size of 500×500 μm^2. Depth profiling analysis was performed by sputtering with a Cs^+ beam (2 keV, 110 nA), and the studied area had a size of

350×350 μm^2. The sputtering rate of the Cs^+ beam was ~0.03 nm s^{-1}. Ti K-edge X-ray absorption near-edge structure (XANES) spectra were collected on the 1W1B beamline of the Beijing Synchrotron Radiation Facility and analyzed by the Athena software (Demeter, Bruce Ravel, New York, USA). Solid-state ^{25}Mg, ^{1}H, and ^{13}C magic-angle-spinning (MAS) nuclear magnetic resonance (NMR) spectroscopy (Avance III, Bruker BioSpin AG, Fällanden, Switzerland) was performed at 14.09 T (600 MHz). The chemical shift of ^{25}Mg was referenced to a 5 M aqueous solution of $MgCl_2$, while the ^{1}H and ^{13}C signals were referenced to tetramethylsilane.

Electrochemical measurements

To fabricate the working electrode, the as-prepared active materials were mixed with Super P and polyvinylidene fluoride (weight ratio: 8:1:1) in *N*-methyl-2-pyrrolidone. The obtained slurry was cast onto a piece of Mo foil at active mass loadings of 1, 2, and 5 mg cm^{-2}. The electrolyte was controlled at 38 μl cm^{-2}, that is, 7.6 μl mg^{-1} at high loadings of 5 mg cm^{-2}. Freshly polished Mg foil pieces were utilized as both the counter and reference electrodes. The APC electrolyte, a 0.4 M 2PhMgCl–$AlCl_3$ solution in tetrahydrofuran (THF), was prepared according to a previously reported procedure involving the reaction of PhMgCl with $AlCl_3$ (2:1) in THF. Coin-type cells were assembled in an Ar-filled glove box. Here, the stacked cathode and anode were separated by a glass fiber membrane (GF/D, Whatman plc (Cytiva), Kent, UK) and immersed into the electrolyte. Cyclic voltammetry (CV) was performed using an electrochemical workstation (CHI660D, CH Instruments Inc., Texas, USA) at a room temperature of 25 °C. Galvanostatic discharge–charge measurements were conducted in the temperature region from −15 °C to 55 °C using a battery testing system (LAND CT2001A, Wuhan Land Co., Ltd. China).

Diffusion coefficient calculations

The galvanostatic intermittent titration technique (GITT) involved the application of constant current pulses for 10 min at 50 mA g^{-1} followed by a 40-min open-circuit voltage relaxation period. The Mg^{2+} diffusion coefficient (D) was calculated under sufficiently low currents over limited time intervals via the following equation: $D = \frac{4}{\pi\tau}\left(\frac{n_M V_M}{S}\right)^2 \left[\frac{\Delta E_s}{\Delta E_\tau}\right]^2$. Here, τ is the duration of the current pulse; n_M and V_M are the molar mass and volume of the material, respectively; and S is the area of the

electrode–electrolyte interface (corresponding to the geometric area of the electrode). Furthermore, ΔEs and $\Delta E\tau$ are the changes in the steady-state voltage and overall cell voltage, respectively, observed after the application of a current pulse in a single-step GITT experiment.

Calculations of energy and power densities

The specific energy of the cell (Es) was calculated via the following equation: $E\text{s} = E \times (C_c^{-1} + C_a^{-1})^{-1}$. Here, E is the discharge voltage (V) representing a mid-point potential (the potential of a cell discharged to 50% of its capacity). Furthermore, C_c is the specific capacity of the cathode (mAh g^{-1}), while C_a is the specific capacity of the Mg anode (2205 mAh g^{-1}). The specific power of the cell (Ps) was calculated via the following equation: $P\text{s} = E\text{s} / t$, where t is the discharge time (h).

Calculation details

Density functional theory (DFT) calculations were performed using the projector augmented wave (PAW) method implemented in the Quantum Espresso software package. Exchange–correlation energies were obtained using the Perdew–Burke–Ernzerhof (PBE) functional. The Kohn–Sham orbitals were expanded in a plane-wave basis with an energy cutoff of 45 Ry and charge density cutoff of 411 Ry. Brillouin-zone integration was performed using the Methfessel–Paxton special point technique with a smearing parameter of 0.05 Ry and k-point meshes of 4×4×1. The titania surface was modeled utilizing an extended four-layer slab system. The Van der Waals (VdW) correction was performed using the DFT–D scheme. To conduct a structural optimization, the entire slab was relaxed until the Hellmann–Feynman forces were lower than 0.001 Ry/a.u. When the energy change per atom was less than 10^{-5} Ry and the mean displacement was less than 0.001 Å, the calculation procedure was converged. The structural models and electron densities were visualized using the XCrySDen software (Anton Kokalj, Slovenia).

The intercalation energies of $MgCl^{+}$ ions were calculated by the described DFT methods via the following equation: $E_{intercalation} = (E_{MgCl+slab} - nE_{MgCl} - E_{slab})/n$. Here, $E_{MgCl+slab}$ is the total energy of the optimized Ti_2O_4 sheet with adsorbed $MgCl^{+}$ species;

E_{MgCl} and E_{slab} are the energies of a single MgCl unit and titanium oxide sheets, respectively; and n is the number of adsorbed $MgCl^+$ ions. The distance between sheets was fixed at 1.1–1.7 nm during calculations.

MgCl structures with and without protons (H_3O^+) were optimized at the DFT level to investigate the stripping of the electrolyte complex. The $MgCl^+$ bond strength was calculated via the following equation: $E_{bond} = E_{MgCl} - E_{Mg} - E_{Cl}$. Here, E_{MgCl} is the energy of the $MgCl^+$ ion, while E_{Mg} and E_{Cl} are the energies of the individual Mg and Cl atoms, respectively. The optimized bond length of the $MgCl^+$ ion was directly measured using a three-dimensional visualization software VESTA (Koichi MOMMA, Japan).

Diffusion barriers of the intercalated Mg and MgCl species in the titanium oxide sheet were determined by a climbing image nudged elastic band method, which optimized a series of intermediate images along the reaction path. Each image located the lowest energy while maintaining equal spacing to the neighboring images. This constrained optimization was performed by adding spring forces between the images along the band and projecting the force component generated by the potential perpendicular to the band. The charge density difference (charge rearrangement) caused by Mg/MgCl intercalation was calculated via the equations $\Delta\rho = (\rho^{Mg+slab} - \rho^{Mg} - \rho^{slab})$ / $\Delta\rho = (\rho^{MgCl+slab} - \rho^{MgCl} - \rho^{slab})$. Charges on various atoms were computed by a Bader charge analysis procedure.

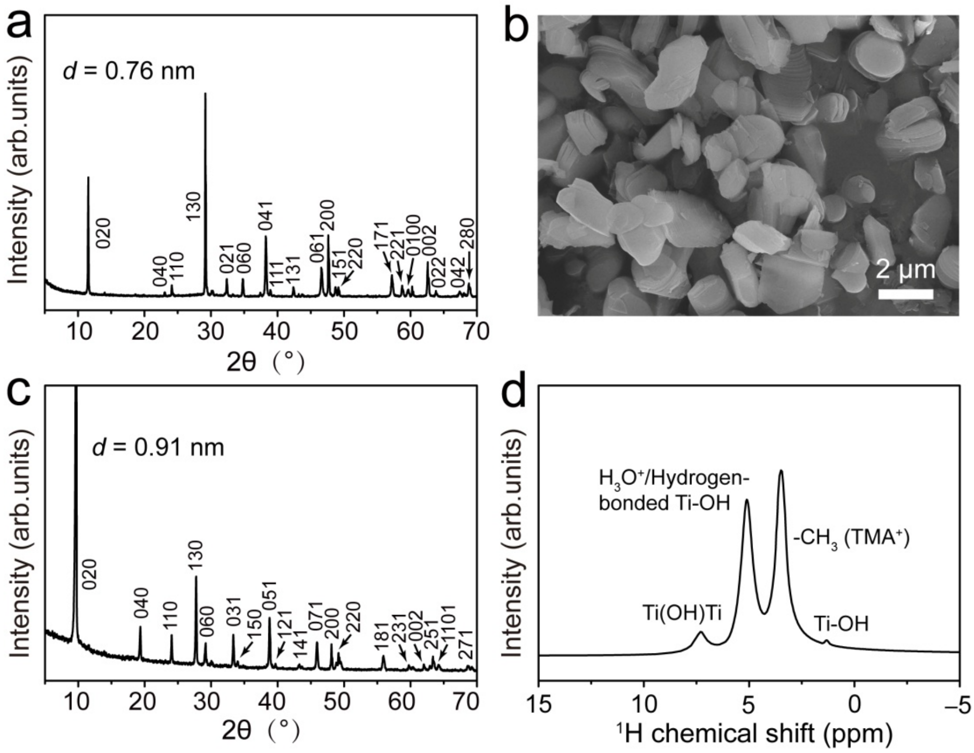

Figure S1. (a) XRD pattern and (b) SEM image of titanate precursor $K_{0.8}[Ti_{1.73}Li_{0.27}]O_4$, showing a micrometer-sized thick-plate morphology. (c) XRD pattern for the sample after proton exchange. The replacement of K^+ with protons brought an increase of interlayer spacing from 0.76 to 0.91 nm, implying that the exchanged protons were in the hydrated form. (d) ^1H solid-state nuclear magnetic resonance (NMR) spectrum collected for freeze-dried sheets. Clear signals corresponding to isolated Ti-OH, hydrogen bonded Ti-OH, H_3O^+, and the OH groups shared by two Ti (Ti(OH)Ti) were observed, suggesting that the protons may exist as hydrated forms of H_3O^+ or being adsorbed on oxygen atoms at the vertexes of the TiO_6 octahedra.

The alkali-metal ions in the interlayer galleries of the layered precursor exhibited ion-exchangeable characteristics; therefore, these ions could be replaced by protons to form a layered protonic titanate $H_{1.07}Ti_{1.73}O_4{\cdot}H_2O$. The subsequent reaction in tetramethylammonium hydroxide (TMAOH) resulted in a swelling of the crystals, and unilamellar $Ti_{1.74}O_4^{1.04-}$ sheets were ultimately obtained after the application of a shear force.

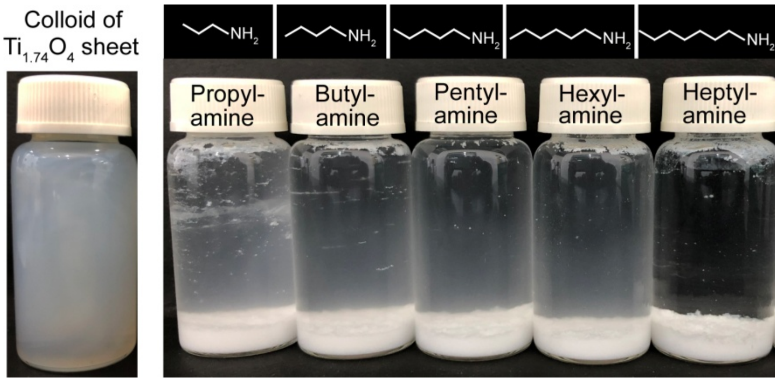

Figure S2. Left: Digital photograph of the pristine suspension of the negatively charged titanium oxide sheets ($Ti_{1.74}O_4^{1.04-}$). Right: Digital photographs of the electrostatic self-assembly containing various protonated amines (propylamine, butylamine, pentylamine, hexylamine, and heptylamine), exhibiting immediate and visible flocculation.

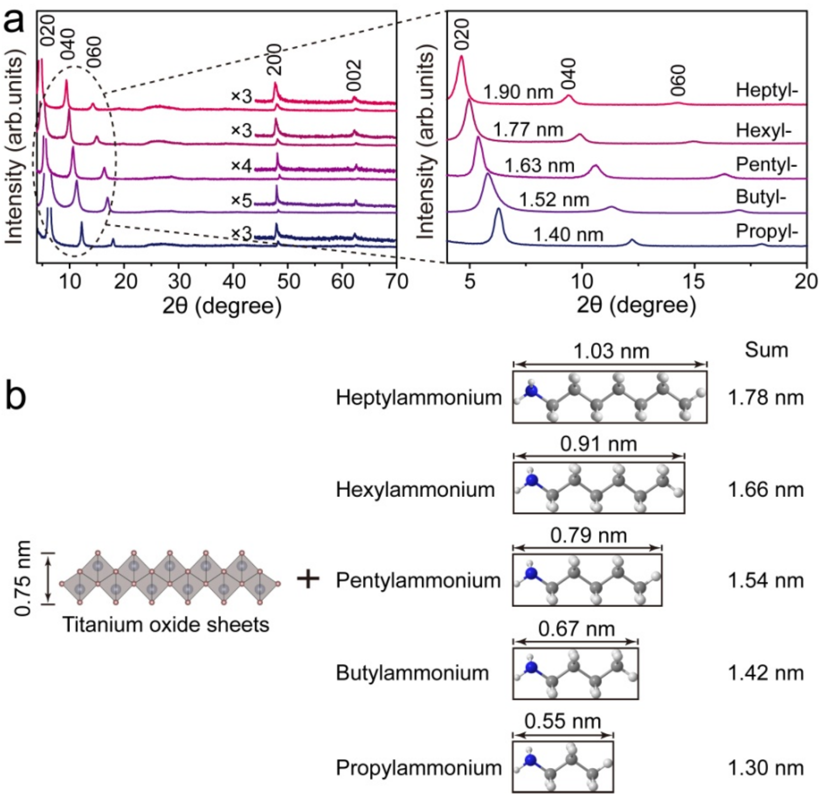

Figure S3. (a) XRD patterns of the titanium oxide sheets coagulated with various alkylammonium ions. The positional changes of the basal reflections are attributed to the different lengths of the alkylammonium ions present in the gallery. All the structures generated two peaks centered at 48.2° and 62.5°, which corresponded to the (200) and (002) intrasheet reflections for the in-plane structure of the titanium oxide sheet, respectively. (b) Various spacings estimated by the addition of the sheet thickness and dimensions of the respective ammonium ions along the axis of the all-trans fully extended alkyl chain (ChemBio 3D® Ultra program). The interlayer gap-dependent (00*l*) stacking reflections shifted toward low angles with an increase in the number of C atoms in the *n*-alkylamine structure. The basal space exhibited a linear increase with increasing number of C atoms, suggesting that alkyl chains were intercalated in a standing conformation with the same tilting angles between the stacked titania sheets. The distance between the layers was almost equal to the sum of the titanium oxide sheet thickness and the sizes of the respective ammonium ions. The mean increase in basal spacing was 0.125 nm, which was close to the length of each CH_2 group along the axis of an all-trans fully extended alkyl chain (0.127 nm). Therefore, alkyl chains were likely arranged as monolayers perpendicularly to the titanate sheets or bilayers with a tilting angle of approximately 30°.

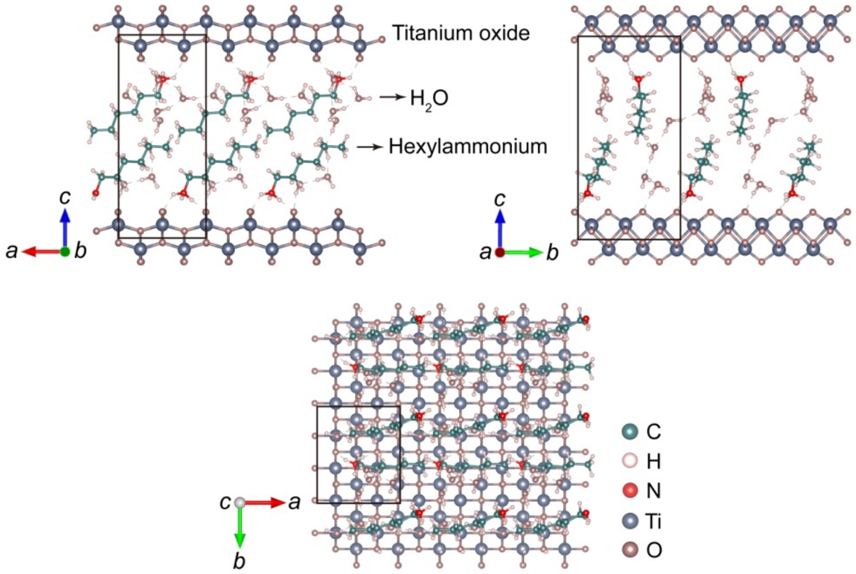

Figure S4. Most stable configuration of the $(Ti_{1.74}O_4)^{1.04-}(C_6H_{16}N^+)_{0.36}(H^+)_{0.68}\cdot 1.37H_2O$ bilayer paraffin-like structure obtained by DFT using the PAW potentials implemented in the Vienna ab initio simulation package (VASP).

The plane wave cutoff was 600 eV. The PBE exchange–correlation functional was employed in all simulations, and the long-range VdW interactions were described by the DFT–D2 method. A 2×3×1 Ti_2O_4 supercell comprising four protons, two $C_6H_{16}N$ molecules, and eight H_2O molecules randomly placed in the gallery between titania sheets, was constructed. The obtained structure was fully optimized using a conjugate gradient method, in which the convergence criterium for the total energy and interaction force were set to 10^{-6} eV and 10^{-3} eV·Å^{-1}, respectively.

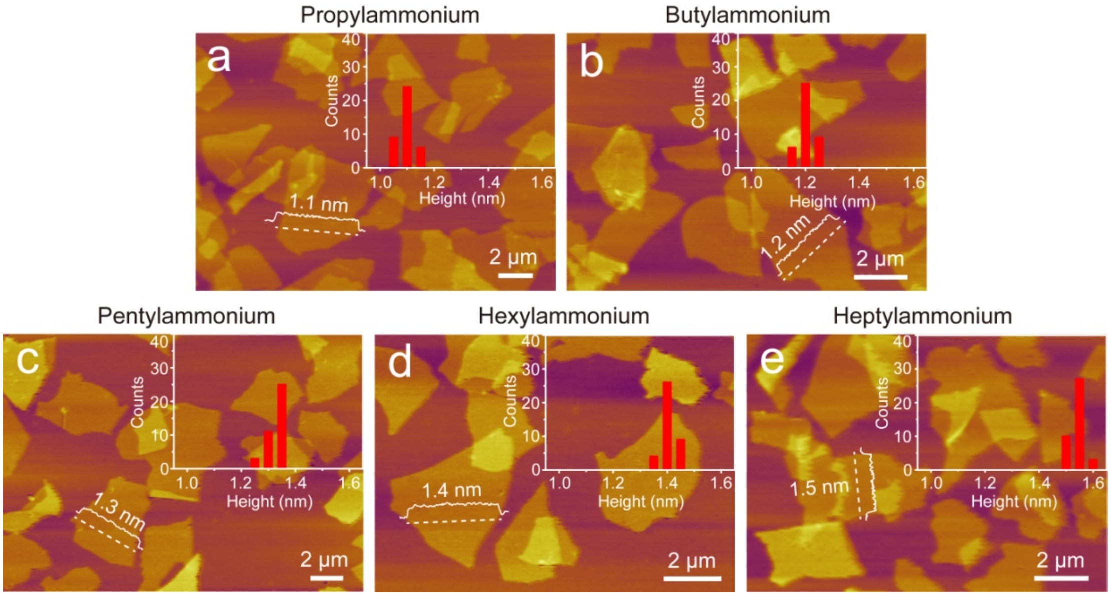

Figure S5. Typical AFM images and statistical analysis of thickness for titanium oxide sheets protonated with various alkylammonium ions. The statistical analysis for the sheet ensemble was performed by randomly selecting ~40 sheets. A step increment was observed for amines with increasing length. For this characterization, the sheets were deposited on a clean Si wafer, dipped into respective aqueous solutions of n-alkylamine for 20 min, and thoroughly washed to remove the weakly absorbed molecules.

a

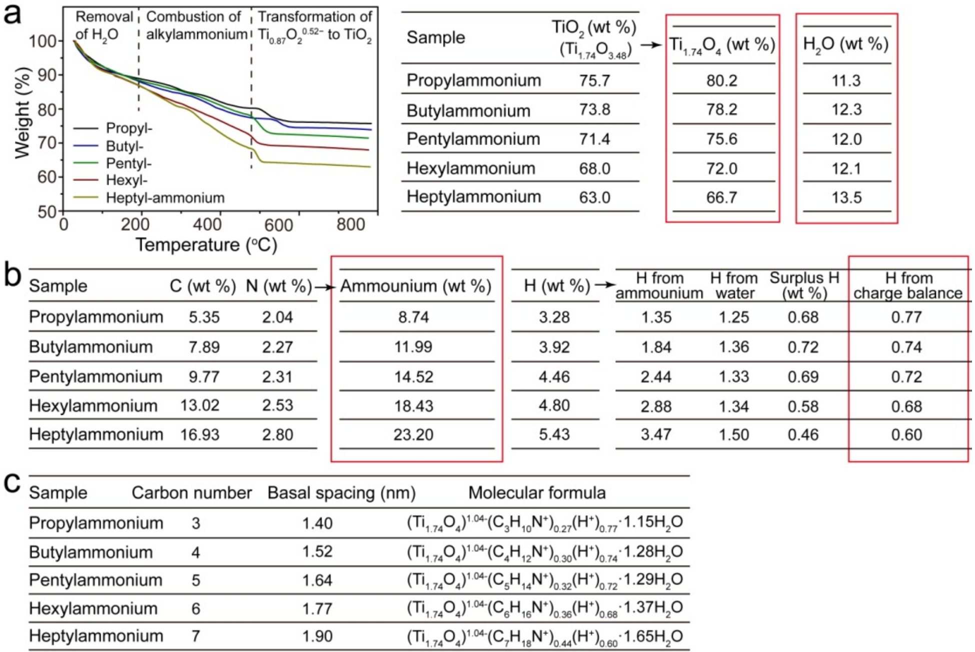

Sample	TiO_2 (wt %) ($Ti_{1.74}O_{3.48}$)	→ $Ti_{1.74}O_4$ (wt %)	H_2O (wt %)
Propylammonium	75.7	80.2	11.3
Butylammonium	73.8	78.2	12.3
Pentylammonium	71.4	75.6	12.0
Hexylammonium	68.0	72.0	12.1
Heptylammonium	63.0	66.7	13.5

b

Sample	C (wt %)	N (wt %) →	Ammounium (wt %)	H (wt %) →	H from ammounium	H from water	Surplus H (wt %)	H from charge balance
Propylammonium	5.35	2.04	8.74	3.28	1.35	1.25	0.68	0.77
Butylammonium	7.89	2.27	11.99	3.92	1.84	1.36	0.72	0.74
Pentylammonium	9.77	2.31	14.52	4.46	2.44	1.33	0.69	0.72
Hexylammonium	13.02	2.53	18.43	4.80	2.88	1.34	0.58	0.68
Heptylammonium	16.93	2.80	23.20	5.43	3.47	1.50	0.46	0.60

c

Sample	Carbon number	Basal spacing (nm)	Molecular formula
Propylammonium	3	1.40	$(Ti_{1.74}O_4)^{1.04-}(C_3H_{10}N^+)_{0.27}(H^+)_{0.77}\cdot 1.15H_2O$
Butylammonium	4	1.52	$(Ti_{1.74}O_4)^{1.04-}(C_4H_{12}N^+)_{0.30}(H^+)_{0.74}\cdot 1.28H_2O$
Pentylammonium	5	1.64	$(Ti_{1.74}O_4)^{1.04-}(C_5H_{14}N^+)_{0.32}(H^+)_{0.72}\cdot 1.29H_2O$
Hexylammonium	6	1.77	$(Ti_{1.74}O_4)^{1.04-}(C_6H_{16}N^+)_{0.36}(H^+)_{0.68}\cdot 1.37H_2O$
Heptylammonium	7	1.90	$(Ti_{1.74}O_4)^{1.04-}(C_7H_{18}N^+)_{0.44}(H^+)_{0.60}\cdot 1.65H_2O$

Figure S6. (a) TGA spectra of the titanium oxide sheets coagulated with various alkylammonium ions that were used to determine the weight fractions of $Ti_{1.74}O_4$ and H_2O species. (b) Results of CHN analyses conducted to determine the contents of ammonium ions and protons. The total weight percentage of titanium oxide, water, and ammonium was approximately 100, indicating high data accuracy. (c) Deduced chemical formulas of the various alkylammonium-spaced specimens. The proton contents were determined using the charge balance equation.

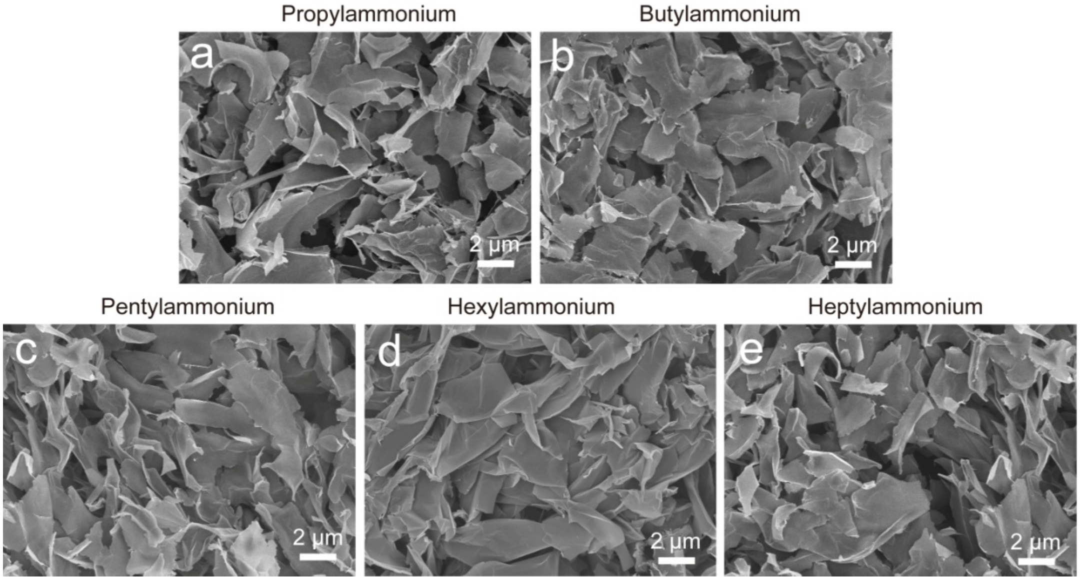

Figure S7. SEM images of the titanium oxide sheets coagulated with varying alkylammonium ions, all showing similar thin platelet morphology.

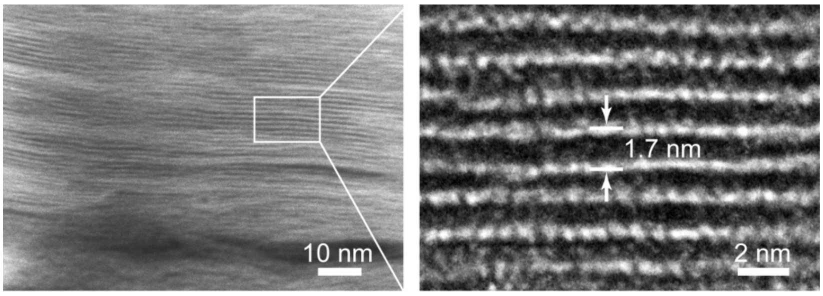

Figure S8. Cross-sectional Cs-corrected HR–TEM images of the hexylammonium-spaced specimen. Distinct lattice fringes with a spacing of 1.7 nm are present in the stacking direction, which is consistent with the XRD results.

Here, the thin cross-sectional lamellae were obtained by a typical in situ lift-out procedure. The lamellae were cut out perpendicularly to the specimen via high-precision site-specific milling (Helios NanoLab 600, FEI Company (Thermo Fisher Scientific), Oregon, USA). The treatment facilitated SEM observation in conjunction with focused ion-beam milling. Pt was deposited using an ion beam to weld the lamellae to a micromanipulator that was afterward lifted from the substrate. The subsequent transfer to a specialized TEM grid (OmniProbe) was followed by gentle polishing with Ga^{+} ions (at 5 kV and 50 pA) to eliminate the side damage and decrease the specimen thickness to 50 nm.

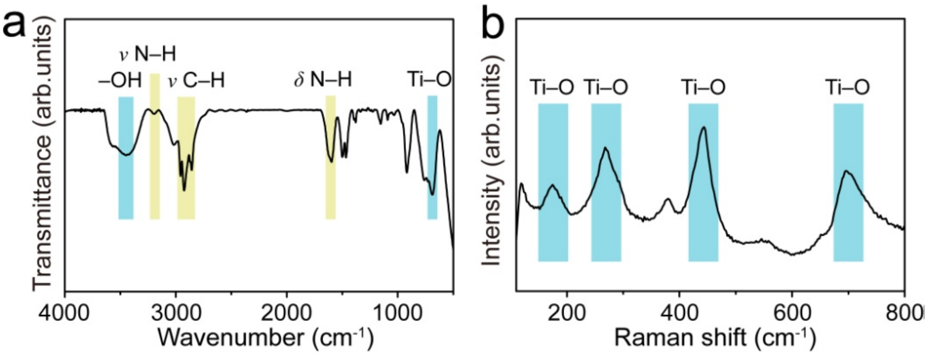

Figure S9. (a) FTIR and (b) Raman spectra of the hexylammonium-spaced titania specimen illustrating the hybridization of titanium oxide and alkylammonium ions.

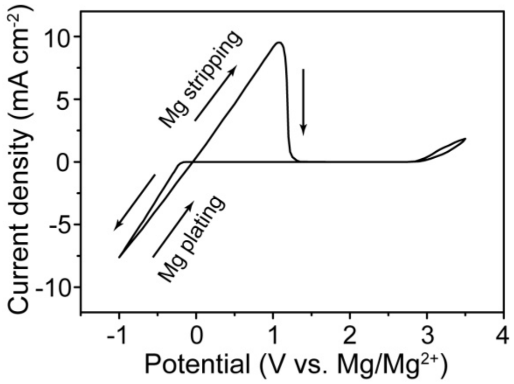

Figure S10. Cyclic voltammograms (CV) profile of the APC electrolyte obtained in a three-electrode system at a scan rate of 25 mV s^{-1}. Mg foil pieces were used as the counter and reference electrodes, while a Pt wire was utilized as the working electrode. The profile demonstrates the electrochemically reversible Mg deposition/dissolution.

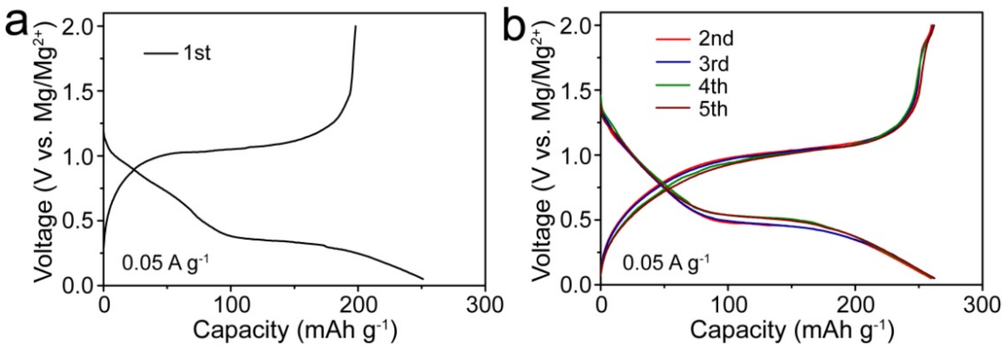

Figure S11. (a) Galvanostatic discharge and charge curves recorded during the first cycle. The initial discharge and charge capacities were 251 mAh g^{-1} (1.37 electron transfers) and 198 mAh g^{-1} (1.08 electron transfers), respectively. These results indicate that some Mg ions remained trapped in the oxide lattice after the initial charging process. As a result, a Coulombic efficiency of approximately 79.0% was achieved. (b) Galvanostatic discharge and charge curves recorded during the subsequent cycles showing the reversible magnesiation and demagnesiation processes. A capacity of 260 mAh g^{-1} (1.42 electron transfers) with a Coulombic efficiency of approximately 100% was achieved.

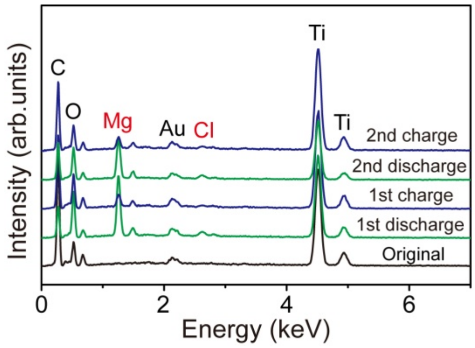

Stage	$Mg:Ti_{1.74}$ atomic ratio (ICP/EDS)	Cl:Mg atomic ratio (EDS)
2nd charge	0.07/0.10	0.12
2nd discharge	0.78/0.78	0.11
1st charge	0.08/0.09	0.13
1st discharge	0.76/0.77	0.12

Figure S12. Results of ex situ EDS elemental analysis and ICP-AES measurements conducted at different discharging and charging stages to determine the Mg contents and Cl-to-Mg ratios of the discharged and charged electrode specimens. There were significant increase and decrease in the Mg content after the completion of the discharge and charge processes, owing the reversible magnesiation and demagnesiation reactions, respectively. The obtained Cl-to-Mg atomic ratio was substantially less than one, suggesting that Mg^{2+} ions were the primary intercalated species.

a

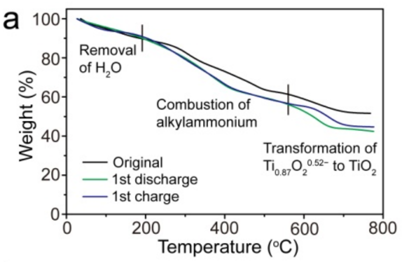

Sample	TiO_2 (wt %) ($Ti_{1.74}O_{3.48}$)	→ $Ti_{1.74}O_4$ (wt %)	H_2O (wt %)
Original	51.6	54.6	8.02
1st discharge	42.4	44.9	6.14
1st charge	44.6	47.2	6.39

b

Sample	C (wt %)	N (wt %) →	Ammounium (wt %)	H (wt %) →	H from ammounium	H from water	Surplus H (wt %)	H from charge balance
Original	25.25	1.90	13.8	3.58	2.17	0.89	0.38	0.68
1st discharge	12.69	0.39	2.84	2.71	0.44	0.68	1.59	0.95
1st charge	13.65	0.40	2.91	2.88	0.45	0.71	1.72	0.95

c

Sample	Ti (wt %)	Mg (wt %)	Cl (wt %)
Original	/	/	/
1st discharge	26.1	5.68	1.02
1st charge	27.2	0.54	0.10

d

Samples	Molecular formula
Original	$(Ti_{1.74}O_4)^{1.04-}(C_6H_{16}N^+)_{0.36}(H^+)_{0.68}\cdot 1.20H_2O$
1st discharge	$(Mg^{2+})_{0.66}(MgCl^+)_{0.10}(Ti_{1.74}O_4)^{1.04-}(C_6H_{16}N^+)_{0.09}(H^+)_{0.95}\cdot 1.11H_2O$
1st charge	$(Mg^{2+})_{0.06}(MgCl^+)_{0.01}(Ti_{1.74}O_4)^{1.04-}(C_6H_{16}N^+)_{0.09}(H^+)_{0.95}\cdot 1.10H_2O$

Figure S13. (a) TGA spectra used to determine the contents of titanium oxide and water species. (b) Results of CHN analyses conducted to determine the contents of interlayer alkylammonium ions and protons. (c) Results of ICP analysis performed to determine the Ti and Mg contents. The Cl content was estimated from the Cl-to-Mg atomic ratio obtained via EDS analysis. (d) Compositions of various titanium oxide electrode samples.

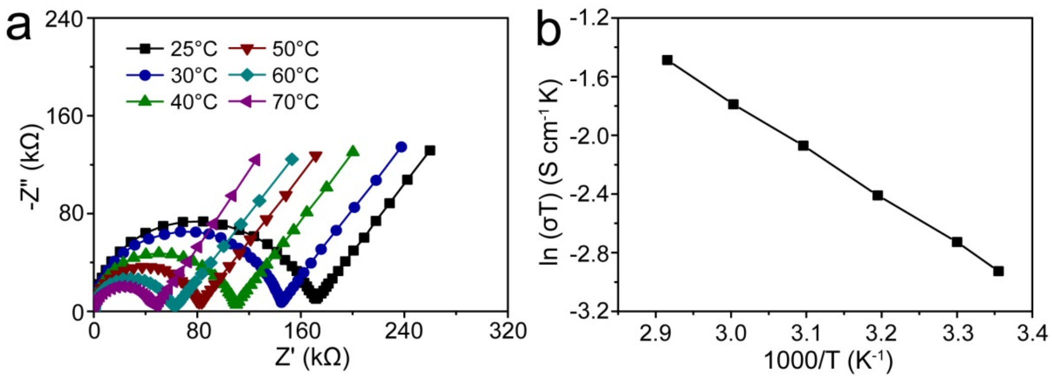

Figure S14. (a) Nyquist plots for prepared titanium oxide sample at temperature ranges of between 25 and 70 °C. (b) Arrhenius plot of Mg-ion conductivity. The Nyquist plots exhibit a semicircle at high frequency and a linear tail at low frequency. The latter is commonly attributed to blocking effects at the electrode and is typical for ionic conductors. The conductivity increased from 1.8×10^{-4} S cm^{-1} at 298 K to 6.6×10^{-4} S cm^{-1} at 343 K. The Arrhenius plot shows a proportional increase in the ionic conductivity with a rise in temperature, yielding E_a = 0.25 eV. This remarkably low activation energy indicating that the obstacles to Mg-ion transport in our material are very small.

The samples was obtained by stirring prepared titanium oxide powder in a APC electrolyte for four days at room temperature, then collected by centrifugation and dried under under 150 °C in vacuum oven for 1day to forming powder. The powders were then added to a standard rectangle die and pressed at 20 Mpa for 30 min, where two opposite surfaces are connected to platinum wires by conductive adhesive paste. The ionic conductivity was measured by electrochemical impedance spectrum (EIS) (Solartron 1260) at different temperature and the frequency range from 1 MHz to 1 Hz with an input voltage amplitude of 100 mV.

The ionic conductivity was calculated from the equation of $\sigma = L/SR$, where σ represents the ionic conductivity, L and S are the length and cross-sectional area of the pressed sample, and R represents the resistance determined by the Nyquist plot. The activation energy (E_a) was calculated using the equation $\sigma = \sigma_0/T \exp(-E_a/kT)$, where σ is the conductivity, σ_0 is the pre-exponential factor, T is absolute temperature, Ea is the activation energy, and k is the Boltzmann constant.

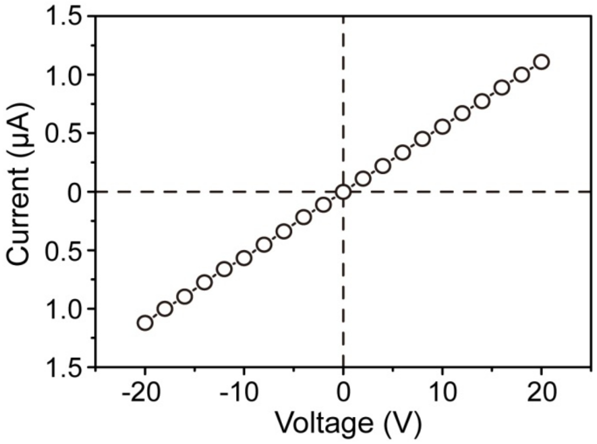

Figure S15. I-V curve collected for the pristine sample of $(Ti_{1.74}O_4)^{1.04-}(C_6H_{16}N^+)_{0.36}(H^+)_{0.68}\cdot 1.37H_2O$ to exclude contribution of electrical conductivity and proton movement. The measurement gave a conductivity of only 4.3×10^{-9} S cm^{-1}, which confirmed a sufficiently electrically insulating behavior and negligible contribution of proton motions.

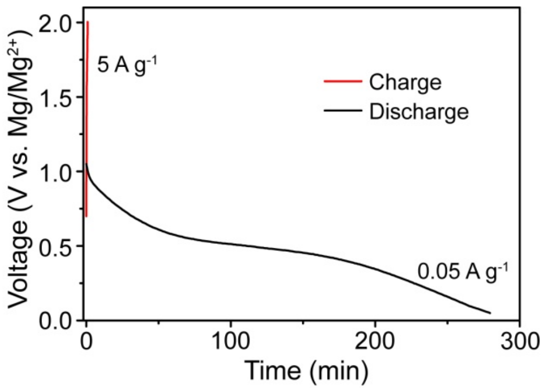

Figure S16. Fast-charging properties of the hexylammonium-spaced electrode. Cell charge at 15 A g^{-1} (47 C) in 55 s and subsequent discharge at 0.05 A g^{-1} (0.16 C) with 4.5 h. The electrode was obtained in a similar slurry preparation manner but with an active material:carbon:binder ratio of 7:2:1. The cells were fully discharged before performing fast charging test.

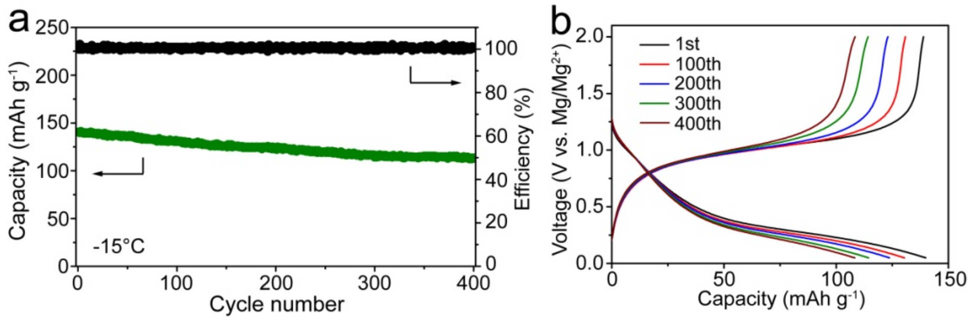

Figure S17. Cycling performance of the hexylammonium-spaced electrode evaluated at a current density of 50 mA g^{-1} and temperature of −15 °C after the three activation cycles conducted at room temperature (25 °C). A capacity of approximately 143 mAh g^{-1} was obtained, and the capacity retention over 400 cycles was 79% (corresponding to a capacity decay of 0.05% per cycle).

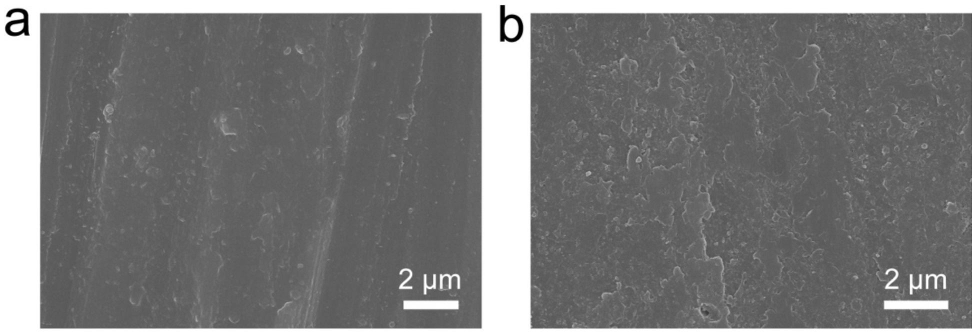

Figure S18. SEM images of the (a) pristine Mg anode before cycling and (b) Mg anode after 500 cycles. No Mg dendrite species were formed.

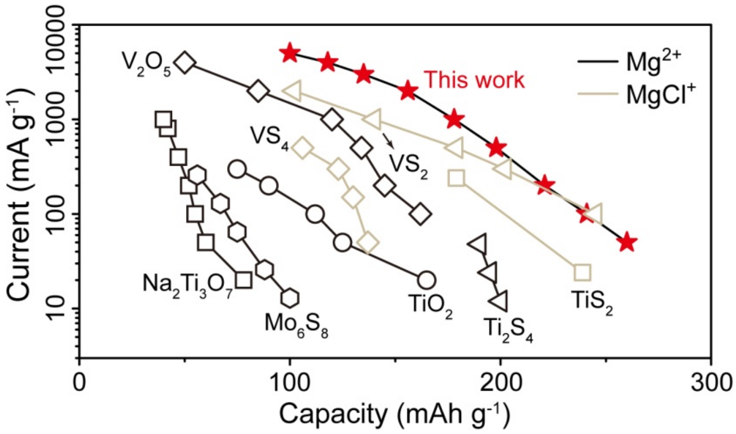

Figure S19. Comparative rate performances of various Mg-ion storage electrodes. The rate performance obtained in the present study was superior to those of the previously reported Mg^{2+} intercalation and even $MgCl^{+}$ intercalation chemical systems.

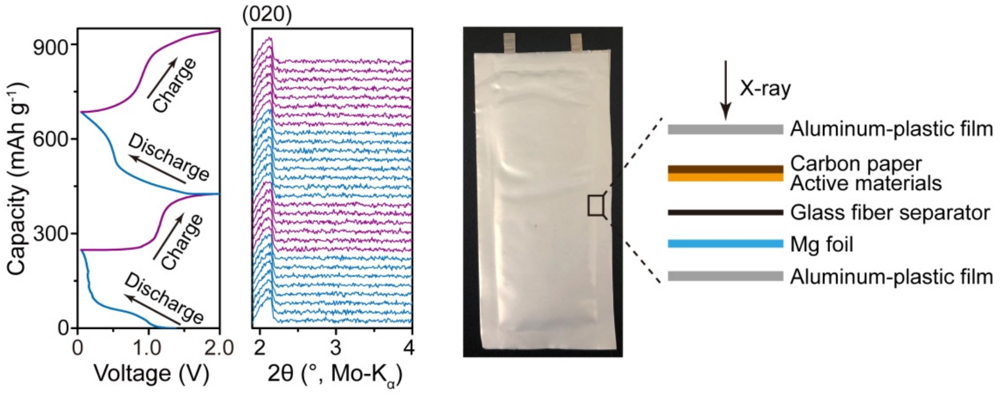

Figure S20. In situ XRD patterns obtained using a pouch cell during the first two cycles conducted at 50 mA g^{-1} in the voltage range between 0.05 and 2.0 V. No new peaks that could be attributed to a phase transformation were detected. Furthermore, the electrochemical magnesiation and demagnesiation processes did not induce any significant deviations of the basal reflections from their original positions, confirming that the host framework underwent a zero-strain insertion.

The pouch cells were assembled inside an Ar-filled glove box (O_2 and H_2O < 0.1 ppm) by stacking the cathode, glass-fiber separator, and anode (Mg foil). C paper was utilized as a current collector instead of Cu or Al foil to ensure high transmittance and prevent corrosion of the electrolyte (0.4 M 2PhMgCl–$AlCl_3$/THF). A laboratory diffractometer with high-intensity Mo-Kα radiation was utilized to record the XRD patterns due to the excellent penetration of the aluminum-plastic foil and C paper by the Mo-Kα beam.

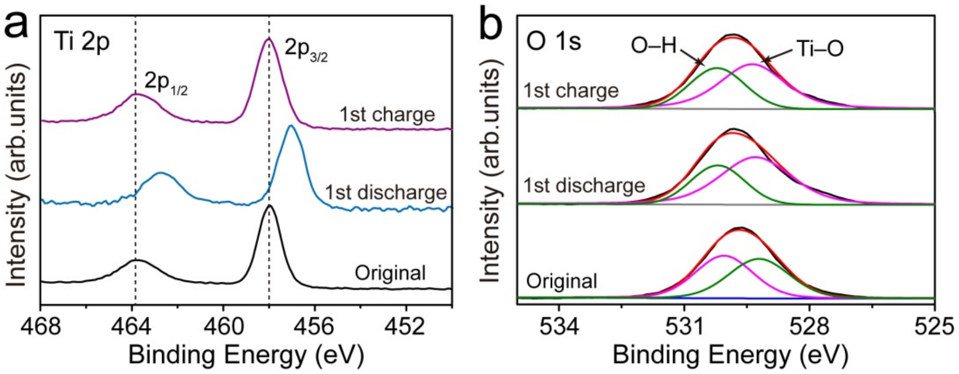

Figure S21. Ex situ high-resolution XPS (a) Ti 2p and (b) O 1s spectra of the electrode specimens subjected to the first full discharge/charge cycle. The Ti 2p spectra exhibit a reversible shift due to the occurrence of electrochemical redox reactions. No visible changes are observed in the O 1s spectra, indicating that O is not involved in the magnesiation or demagnesiation reactions.

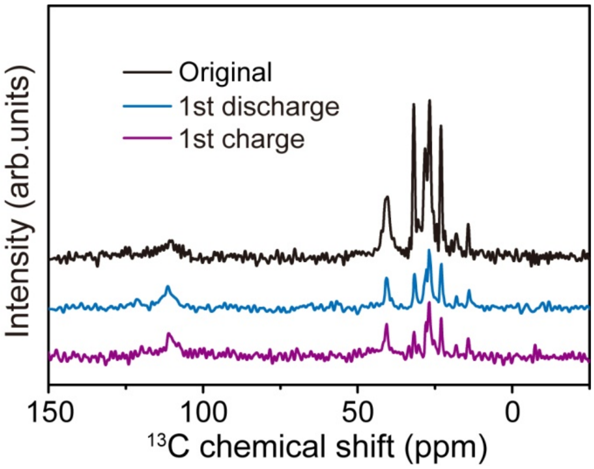

Figure S22. ^{13}C MAS NMR spectra of the pristine, fully discharged, and fully charged electrode specimens. The absence of visible differences in the spectra indicates that THF solvent molecules do not contribute to the high electrode capacity.

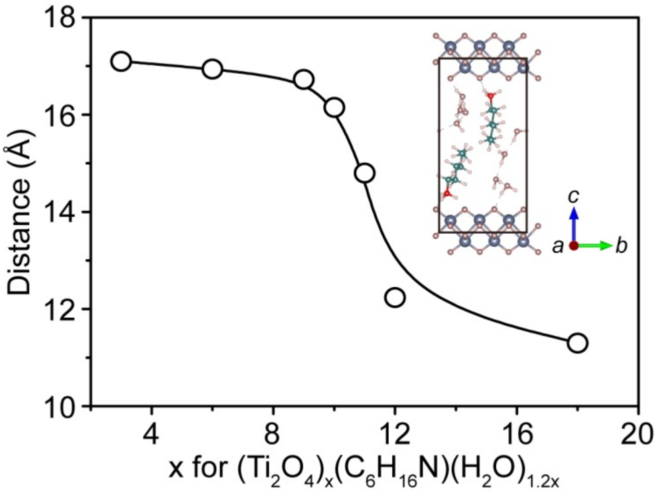

Figure S23. Results of DFT calculations that were performed to determine the amount of hexylammonium ions required to maintain the pillaring structure. The residual amount of $C_6H_{16}N$:$Ti_{1.74}O_4$ (= 0.09:1) is sufficient to stabilize the stacking structure with a high interlayer distance of 1.7 nm. In these simulations, the number of $C_6H_{16}N$ units was fixed at one, while the amount of Ti_2O_4 was varied to obtain different $C_6H_{16}N$:Ti_2O_4 ratios. The amount of H_2O was 1.2 times greater than the Ti_2O_4 amount.

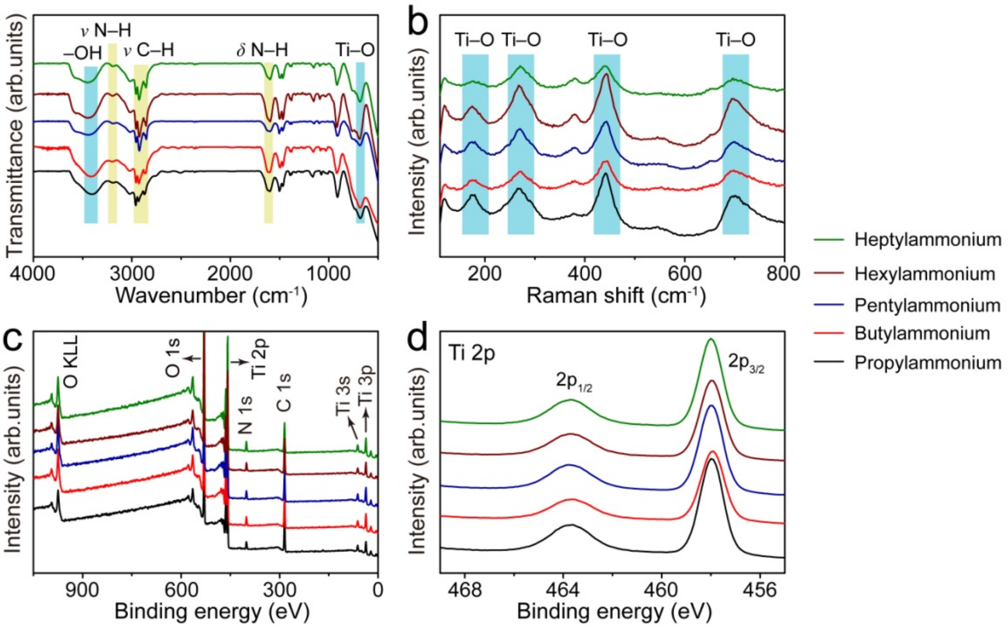

Figure S24. Characterization of various alkylammonium-spaced specimens. (a) FTIR, (b) Raman, (c) XPS survey, and (d) XPS high-resolution Ti 2p spectra. All specimens exhibit similar structural characteristics with the hybridization of titanium oxide and alkylammonium ions. However, their interlayer distances are different due to the different lengths of the spacers. The propylammonium-, butylammonium-, pentylammonium-, hexylammonium-, and heptylammonium-spaced specimens with interlayer distances of 1.40, 1.52, 1.63, 1.77, and 1.90 nm demonstrated Mg intercalation capacities of 0.35, 0.52, 0.66, 0.71, and 0.69 per $Ti_{1.74}O_4$ unit, respectively

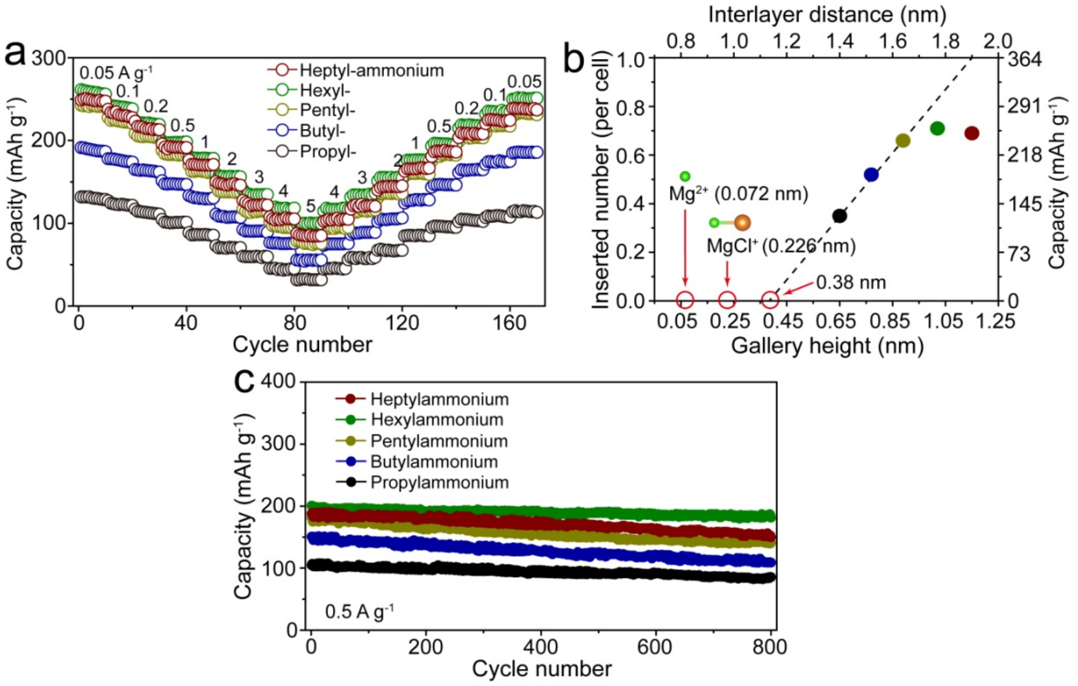

Figure S25. (a) Rate capabilities of the titanium oxide sheets coagulated with various alkylammonium ions. (b) Correlation plot of the gallery height (interlayer distance − sheet thickness) versus the number of inserted Mg^{2+} ions per cell. It shows a strong dependence of the storage capacity on the gallery height. A linear function is observed for the propylammonium-, butylammonium-, and pentylammonium-spaced specimens. A critical gallery height of 0.38 nm was obtained from the intercept of the graph extension with the abscissa axis. (c) Long-term cycling performances of the electrode specimens. For samples with even longer pillars, only a slight performance drop was observed. This could be because the diffusion of Mg^{2+} mainly occurred in the in-plane direction.

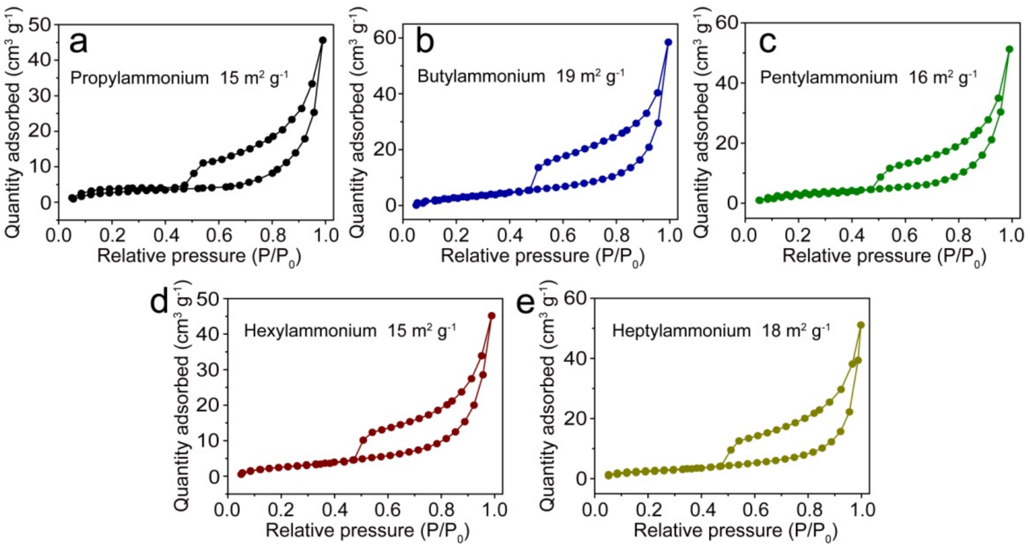

Figure S26. N_2 adsorption–desorption isotherms of the electrode specimens coagulated with various alkylammonium ions. All isotherms show comparable results.

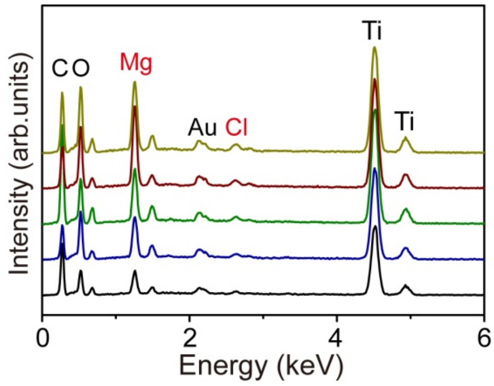

Samples	Cl:Mg atomic ratio (EDS)
Heptylammonium	0.13
Hexylammonium	0.12
Pentylammonium	0.11
Butylammonium	0.12
Propylammonium	0.14

Figure S27. EDS spectra and the corresponding Cl-to-Mg ratios obtained for the titanium oxide sheets spaced with various alkylammonium ions. The calculated atomic ratios are substantially less than one, indicating that Mg^{2+} ions are the primary intercalated species.

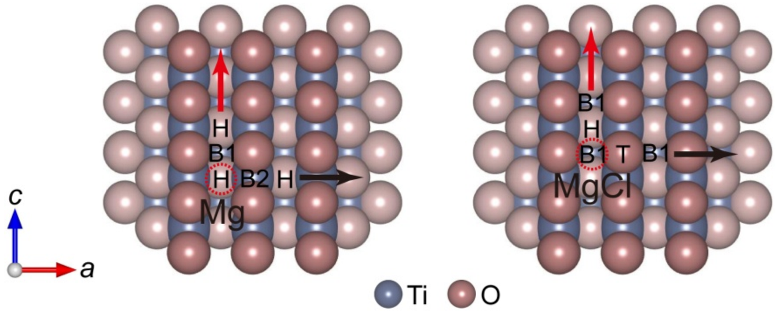

Figure S28. Selected sites and paths for the diffusion barrier calculations. Individual species such as Mg and MgCl are separately placed on the marked high-symmetry sites of the slab surface to determine their global minimum structures. Geometry optimization results revealed that the hollow (H) and long-bridge (B1) sites were the minimum energy sites for Mg and MgCl, respectively.

For Mg diffusion, single Mg atoms were placed at the hollow (H) sites to simulate the initial and final states without any constraints. The diffusion pathway lied either along (H–B1–H) or perpendicularly (H–B2–H) to the atomic trough. For MgCl diffusion, the molecular species were positioned at two successive long-bridge (B1) sites as the intial and final images for the surface diffusion. Therefore, the two pathways in the orthogonal directions (B1–H–B1 and B1–T–B1) were considered to examine the anisotropy of surface diffusion.

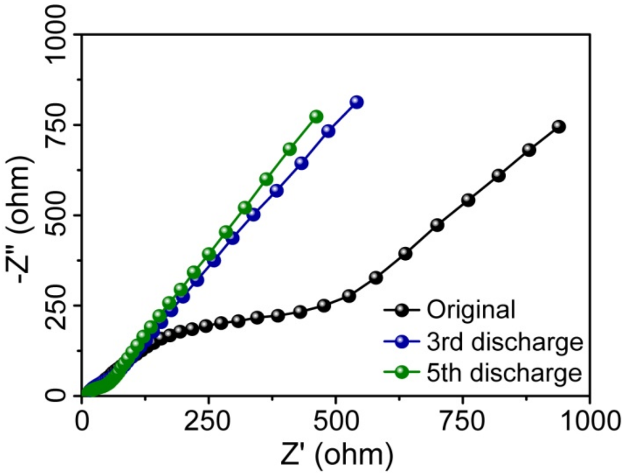

Figure S29. Nyquist plots of the hexylammonium-spaced titania electrodes obtained before and after cycling at 50 mA g^{-1}. The semicircle in the high-medium frequency region indicates the charge-transfer resistance (R_{ct}) at the electrode–electrolyte interface. The significant decrease in R_{ct} after Mg^{2+} intercalation suggests that the latter substantially increases the electrical conductivity of titanium oxide. Therefore, the intercalation process promotes fast electron transport.

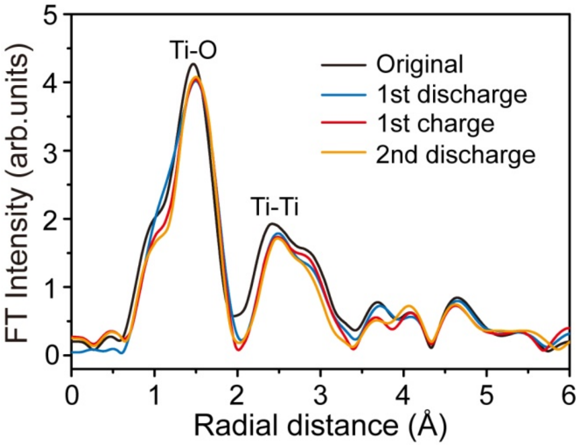

Figure S30. Fourier-transformed extended X-ray absorption fine structure spectra recorded at the Ti K-edge of the designed titanium oxide electrode in different discharge/charge states.

The pristine specimen exhibits two distinct peaks at 1.4 Å and 2.4 Å corresponding to the Ti–O and Ti–Ti bonds, respectively. The amplitudes of the two peaks slightly decrease after the first discharge process; furthermore, the peak position after the first discharge shifts to a higher radial distance as compared with that in the pristine state, which indicates a higher degree of the structural distortion caused by the Mg insertion into the host lattice. The amplitudes of the Ti–O and Ti–Ti bonds remain approximately identical in the subsequent cycles, indicating minor changes in the electrode structure during magnesiation/demagnesiation. The structural distortion facilitates Mg^{2+} diffusion, and the high bonding stability during the reaction cycles contributes to the excellent cycling performance.

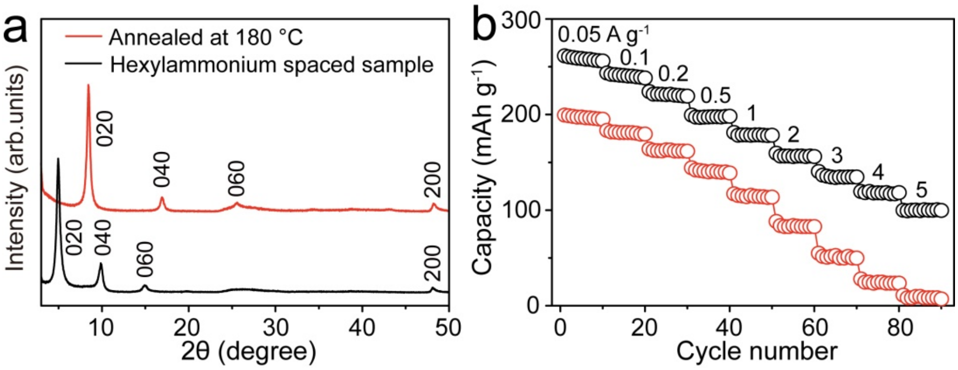

Figure S31. (a) XRD spectra and (b) electrochemical Mg-ion storage characteristics of the electrode obtained before and after thermal annealing at 180 °C. After dehydration, a decrease in the basal spacing from 1.77 to 1.04 nm was accompanied by the electrode performance degradation.

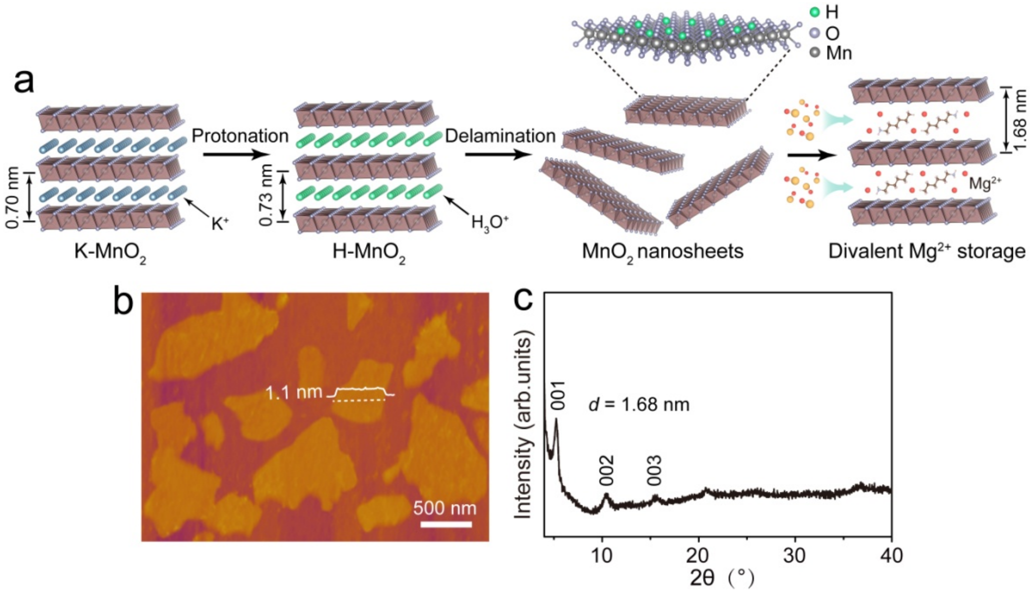

Figure S32. Hexylammonium ion-spaced H_x-MnO_2 sheets for Mg^{2+} storage. (a) Schematic illustration of the synthesis of H_x-MnO_2 sheets and Mg^{2+} intercalation process. (b) AFM image of the H_x-MnO_2 sheets. (c) XRD patterns of the hexylammonium-spaced MnO_2 specimen with a high interlayer distance of 1.68 nm.

Preparation of MnO_2 nanosheets. MnO_2 nanosheets were synthesized using a previously developed method (*Chem. Mater*. **2007**, 19, 6504). Layered K-birnessite was prepared as the starting material via the redox reaction between Mn^{2+} and MnO_4^- ions. A $KMnO_4$–$MnCl_2$–KOH mixture with a Mn^{2+}/MnO_4^- molar ratio of 2 was subjected to a hydrothermal reaction. For this purpose, 0.036×10^{-3} M $KMnO_4$ and 2.14×10^{-3} M KOH solutions were mixed and vigorously stirred to achieve homogeneity. After that, 1.0 M aqueous $MnCl_2$ solution was added to the resulting hot and dark-green solution. A black slurry was obtained via continuous stirring for 2 h and subsequent cooling. This slurry was transferred into a Teflon-lined stainless-steel autoclave for a hydrothermal treatment conducted at 175 °C for 2 d. The obtained product was rinsed with water to remove the KOH residue and subsequently air-dried to form a dark-gray K-birnessite solid. This solid was stirred with a 0.5 M aqueous solution of ammonium persulfate $(NH_4)_2S_2O_8$) at 60 °C for 12 h and then with a 1 M HCl solution at 25 °C for 2 h to form the protonated H-birnessite phase. Finally, the as-prepared H-birnessite was immersed in an aqueous solution of TMAOH and shaken at 120 rpm for 2 d. As a result, a colloidal suspension of H_x-MnO_2 sheets was obtained.

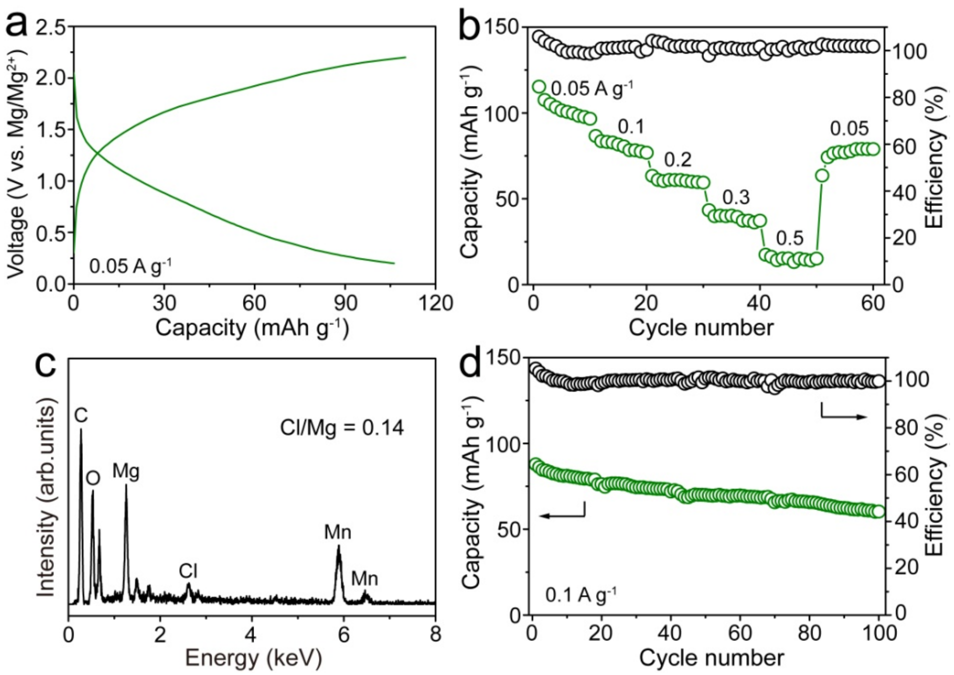

Figure S33. Electrochemical performance of the hexylammonium-spaced MnO_2 specimens. (a) Galvanostatic discharge/charge profiles and (b) rate performance of the cell obtained at charge/discharge current densities of 0.05–0.5 A g^{-1}. (c) EDS spectrum of the fully discharged electrode. The obtained Cl-to-Mg atomic ratio is 0.14, indicating almost exclusive Mg^{2+} storage. (d) Cycling performance at a current density of 0.1 A g^{-1}.

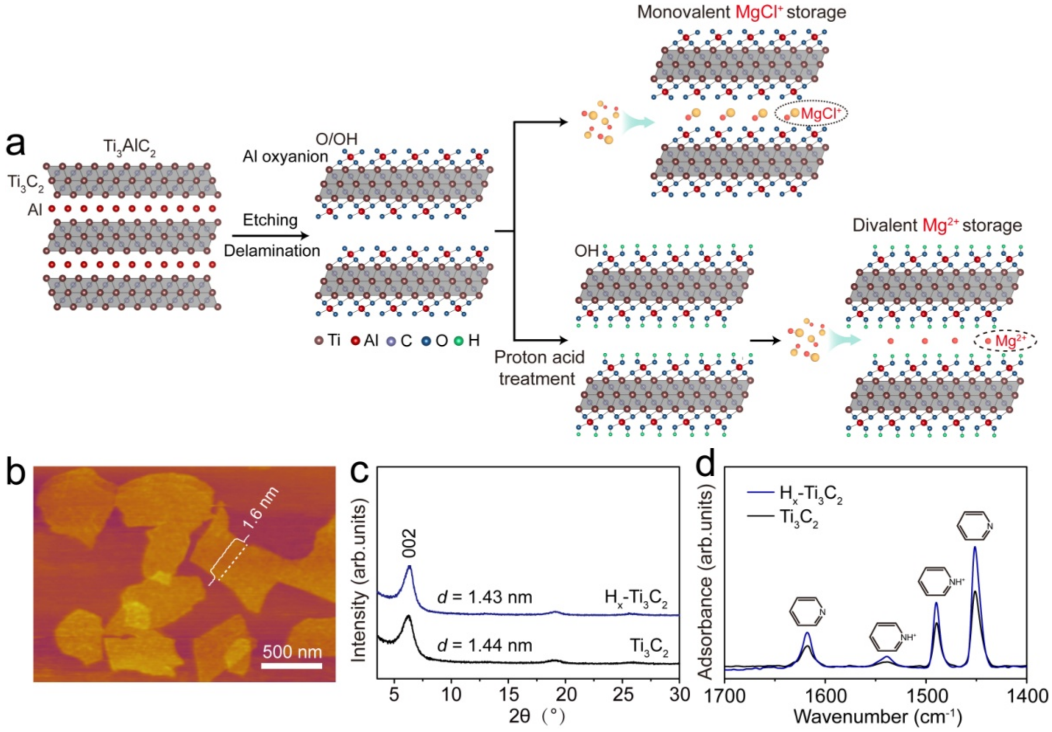

Figure S34. Characterizations of the heptylammonium-spaced oxyanion-terminated Ti_3C_2 specimens. (a) Schematic illustration of the synthesis of H_x-Ti_3C_2 sheets and Mg^{2+} intercalation process. (b) AFM image of the exfoliated Ti_3C_2 sheets. (c) XRD patterns of the heptylammonium-spaced Ti_3C_2 specimens with/without proton acid preprocessing. No visible changes in the XRD pattern were observed after proton implantation, which indicated not only the absence of a phase transformation but also possible adsorption of protons on Ti_3C_2 sheets. (d) FT–IR spectra of the pyridine molecules adsorbed on the H_x-Ti_3C_2 and Ti_3C_2 samples at 50 °C. The bands corresponding to pyridinium ions formed through the bonding of pyridine with Brønsted acid sites are prominent, and the integrated area for the H_x-Ti_3C_2 sample is 1.15 times larger than that for the Ti_3C_2 sample.

Preparation of Ti_3C_2 nanosheets. The colloidal suspension of Ti_3C_2 sheets was prepared using our previously developed method (*Angew. Chem., Int. Ed.* 2016, 55, 14569). Initially, Ti_3AlC_2 powder was pretreated with a diluted HF solution (10 wt%). Subsequently, the crystals were immersed in a 25 wt.% TMAOH solution and kept there for 24 h to hydrolyze the interlayer Al atoms. The slurry obtained after the etching process was washed with deionized water via repeated centrifugation until the solution pH was approximately 6. The sediment of the centrifuge, i.e., multilayer Ti_3C_2, was dispersed in deionized water prior to sonication for 40 min in an ice bath

with Ar bubbling. The sonication solution was centrifuged at 3,500 rpm for 1 h. Finally, the colloidal solution of Ti_3C_2 in water was collected as a dark supernatant.

Preparation of proton-modified Ti_3C_2. The delaminated Ti_3C_2 dispersion (1 mg mL^{-1}) was acidulated with a low-concentration HCl solution (0.0001 M) under stirring.

Coagulation of sheets with ammonium ions. The heptylammonium-spaced Ti_3C_2 specimens were prepared by mixing the aqueous dispersion of Ti_3C_2 or H_x-Ti_3C_2 nanosheets with an aqueous solution of heptylamine. Initially, the heptylamine solution (0.5 mL) was diluted to 40 mL with water. Thereafter, the Ti_3C_2 or H_x-Ti_3C_2 nanosheet dispersion (60 mL, 1 mg mL^{-1}) was added dropwise to the heptylamine solution under constant stirring at a room temperature of 25 °C. The mixing process immediately induced flocculation in the beaker. After overnight stirring, the formed precipitate was collected via repeated centrifugation and thorough washing with pure H_2O. Finally, the desired powder was obtained via freeze drying.

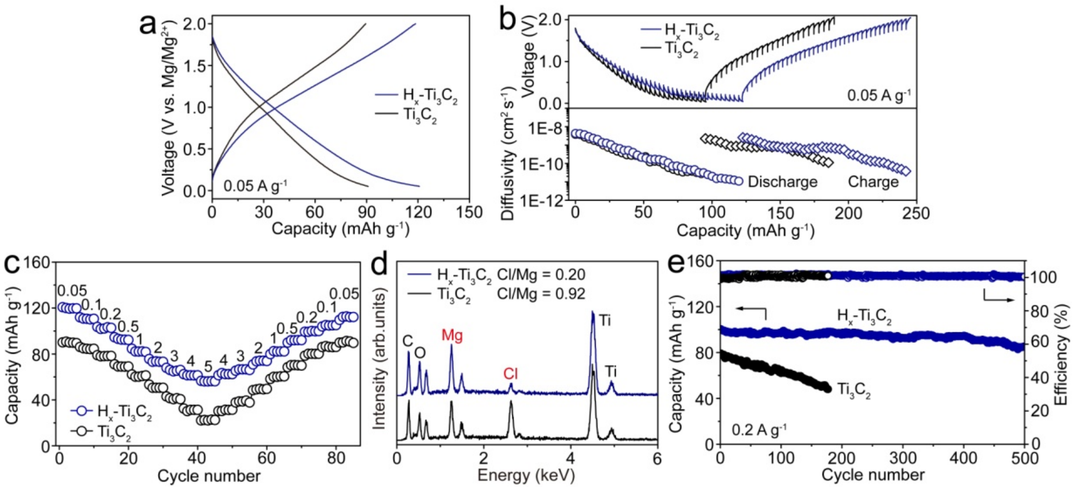

Figure S35. Electrochemical performances of the heptylammonium-spaced H_x-Ti_3C_2 and non-protonated specimens. (a) Galvanostatic discharge/charge profiles. (b) GITT curves recorded for the Ti_3C_2 and H_x-Ti_3C_2 electrodes with the corresponding $MgCl^+$ and Mg^{2+} diffusion coefficients. The calculated diffusion coefficient for Mg^{2+} intercalation is comparable to that determined for $MgCl^+$ intercalation. (c) Rate performance evaluated at charge/discharge current densities of 0.05–5 A g^{-1}. (d) EDS spectra of the fully discharged electrode. The Cl-to-Mg atomic ratio significantly decreased to 0.20 after proton acid preprocessing, which confirmed the effect of surface protons on the dissociation of the Mg–Cl ion pair. The presence of protons efficiently lowered the desolvation energy penalty and significantly increased the proportion of divalent Mg^{2+} ions. (e) Cycling performance evaluated at a current density of 0.2 A g^{-1}.

Table S1. Comparative ion conductivity of our titanium oxide with other oxides and known excellent Mg-ion conducting materials. Due to the strong interaction of Mg^{2+} with O, a high temperature of >500 °C is typically essential for enabling Mg^{2+} transportation in oxides. Comparing with all reports as far as we know, this is still the highest record, even magnitudes higher than excellent ion-conducting MOF materials and on the same order as polymer electrolyte.

**Types**	**Materials**	**Ionic conductivity (S cm^{-1})**	**Temperature**
Oxides	**This work**	**1.8×10^{-4}**	**25 °C**
	$MgHf(WO_4)_3$[1]	2.5×10^{-4}	600 °C
	$Mg_{0.5}Zr_2(PO_4)_3$[2]	7.1×10^{-5}	500 °C
	$Mg_{0.6}Al_{1.2}Si_{1.8}O_6$[3]	2.3×10^{-6}	500 °C
Polymer electrolyte	$Mg(BH_4)_2$-based polymer electrolyte[4]	4.76×10^{-4}	25 °C
	$Mg(ClO_4)_2$-based polymer electrolyte[5]	1.1×10^{-4}	30 °C
MOFs	Cu-azolate MOF[6]	1.3×10^{-4}	25 °C
	Mg-MOF-74[7]	3.17×10^{-6}	25 °C
	Cu-azolate MOF[8]	8.8×10^{-7}	25 °C
Hydrides	$Mg(BH_4)(NH_3BH_3)_2$[9]	1.3×10^{-5}	30 °C
	$Mg(BH_4)(NH_2)$[10]	1.0×10^{-6}	150 °C

Table S2. Comparative electrochemical performances of titanium oxide and the previously reported inorganic electrode materials.

**Storage ion**	**Material**	**Current density (mA g^{-1})**	**$C_{cathode}$ (mAh g^{-1})**	**Charge time (min)**	**P_{Max} (W kg^{-1})**
**Mg^{2+}**	**This work (RT)**	**15000**	**210**	**1**	**7400**
	Mo_6S_8 (RT)[11]	258	56	13	231
	Mo_6S_8 (50 °C)[11]	258	94	22	270
	Ti_2S_4 (60 °C)[12]	48	190	237	51
	Anatase TiO_2 (RT)[13]	300	75	15	203
	$Na_2Ti_3O_7$ (RT)[14]	1000	40	2.4	785
**$MgCl^+$**	$VOPO_4$ (RT)[15]	2000	109	3.3	953
	TiS_2 (RT)[16]	240	179	45	155
	TiS_2 (60 °C)[16]	480	400	50	325
	VS_2 (RT)[17]	2000	102	3	1911
	VS_4 (RT)[18]	2000	86	2.6	2117

Table S3. Comparative cycling performances of titanium oxide and other Mg-ion storage systems.

Material	Electrode configuration	Electrolyte	Anode	Current density	Cycling stability
**This work**	**Two-electrode coin-type cells**	**$PhMgCl-AlCl_3/THF$**	**Mg**	**500 mA g^{-1}**	**81% after 2000 cycles**
Mo_6S_8[11]	Two-electrode coin-type cells	(*tert*-$BuOMgCl)_6$-$AlCl_3/THF$	Mg	13 mA g^{-1}	92% after 40 cycles
Ti_2S_4[12]	Two-electrode coin-type cells	$PhMgCl-AlCl_3/THF$	Mg	24 mA g^{-1}	86% after 40 cycles
Anatase TiO_2[13]	Two-electrode coin-type cells	$PhMgCl-AlCl_3/THF$	Mg	300 mA g^{-1}	80% after 500 cycles
$Na_2Ti_3O_7$[14]	Two-electrode coin-type cells	$PhMgCl-AlCl_3/THF$	Mg	200 mA g^{-1}	96% after 500 cycles
$VOPO_4$[15]	Two-electrode coin-type cells	$PhMgCl-AlCl_3/THF$	Mg	100 mA g^{-1}	70% after 500 cycles
TiS_2[16]	Two-electrode coin-type cells	$PhMgCl-AlCl_3/THF$	Mg	240 mA g^{-1}	80% after 400 cycles
VS_2[17]	Two-electrode coin-type cells	$Mg(HMDS)_2$-$4MgCl_2/THF$	Mg	1000 mA g^{-1}	70% after 600 cycles
VS_4[18]	Two-electrode coin-type cells	$PhMgCl-AlCl_3/THF$	Mg	500 mA g^{-1}	63% after 200 cycles
MoS_2[19]	Three-electrode cells	$PhMgCl-AlCl_3/THF$	Mg	5 mA g^{-1}	94% after 30 cycles
NaV_3O_8[20]	Two-electrode coin-type cells	$PhMgCl-AlCl_3/THF$	Activated carbon cloth	500 mA g^{-1}	88.3% after 100 cycles
TiO_{2-x}[21]	Two-electrode coin-type cells	$PhMgCl-AlCl_3/THF$	Mg	300 mA g^{-1}	76% after 400 cycles
$TiSe_2$[22]	Two-electrode coin-type cells	$Mg(AlCl_2EtBu)_2$/ THF	Mg	5 mA g^{-1}	72% after 50 cycles
α-V_2O_5[23]	Two-electrode coin-type cells	$Mg(TFSI)_2$/ PY14TFSI	Activated carbon cloth	C/10	76% after 50 cycles
ζ-V_2O_5[24]	Two-electrode coin-type cells	$Mg(TFSI)_2/PC$	Activated carbon cloth	6 mA g^{-1}	64% after 50 cycles
$Cu_{2-x}Se$[25]	Two-electrode coin-type cells	$Mg(HMDS)_2$-$AlCl_3$/ diglyme	Mg	1000 mA g^{-1}	84% after 500 cycles
CuS[26]	Two-electrode coin-type cells	$Mg(ClO_4)_2/AN$	Activated carbon cloth	20 mA g^{-1}	75% after 20 cycles
$Na_3V_2(PO_4)_3$[27]	Three-electrode cells	$Mg(BF_4)_2$ in EC/DEC	Activated carbon/Ag wire	5 mA g^{-1}	79% after 20 cycles

Abbreviations: THF, tetrahydrofuran; PC, propylene carbonate; AN, acetonitrile; EC, ethylene carbonate; DEC, diethyl carbonate; $Mg(HMDS)_2$, magnesium bis(hexamethyldisilazide); $Mg(TFSI)_2$, magnesium bis(trifluoromethylsulfonyl)imide; PY14TFSI, 1-butyl-1-methylpyrrolidinium bis(trifluoromethylsulfonyl)imide

References

1. Omote, A., Yotsuhashi, S., Zenitani, Y. & Yamada, Y. High ion conductivity in $MgHf(WO_4)_3$ solids with ordered structure: 1-D alignments of Mg^{2+} and Hf^{4+} ions. *J. Am. Ceram. Soc*. **94**, 2285–2288 (2011).

2. Anuara, N. K., Adnana, S. B. R. S. & Mohamed, N. S. Characterization of $Mg_{0.5}Zr_2(PO_4)_3$ for potential use as electrolyte in solid state magnesium batteries. *Ceram. Int*. **40**, 13719–13727 (2014).

3. Takeda, H., Nakano, K., Tanibata, N. & Nakayama, M. Synthesis, crystal structure and ionic conductivity of $MgAl_2X_8$ (X = Cl, Br). *Sci. Technol. Adv. Mater*. **21**, 131−138 (2020).

4. Du, A. et al. A crosslinked polytetrahydrofuran-borate-based polymer electrolyte enabling wide-working-temperature-range rechargeable magnesium batteries. *Adv. Mater*. **31**, 1805930 (2019).

5. Ramaswamy, M. et al. Magnesium ion conducting polyvinyl alcohol–polyvinyl pyrrolidone-based blend polymer electrolyte. *Ionics* **23**, 1771–1781 (2017).

6. Miner, E. M., Park, S. S. & Dincă, M. High Li^+ and Mg^{2+} conductivity in a Cu-azolate metal−organic framework. *J. Am. Chem. Soc*. **141**, 4422−4427 (2019).

7. Luo, J. et al. A metal–organic framework thin film for selective Mg^{2+} transport. *Angew. Chem. Int*. Ed. **58**, 15313–15317 (2019).

8. Park, S. S., Tulchinsky, Y. & Dincă, M. Single-ion Li^+, Na^+, and Mg^{2+} solid electrolytes supported by a mesoporous anionic Cu−azolate metal−organic framework. *J. Am. Chem. Soc*. **139**, 13260−13263 (2017).

9. Kisu, K. et al. Magnesium borohydride ammonia borane as a magnesium ionic conductor. *ACS Appl. Energy Mater*. **3**, 3174−3179 (2020).

10. Higashi, S., Miwa, K., Aoki, M. & Takechi, K. A novel inorganic solid state ion conductor for rechargeable Mg batteries. *Chem. Commun*., **50**, 1320-1322 (2014).

11. Liao, C. et al. Highly soluble alkoxide magnesium salts for rechargeable magnesium batteries. *J. Mater. Chem. A* **2**, 581-584, (2014).

12. Sun, X. et al. A high capacity thiospinel cathode for Mg batteries. *Energy Environ. Sci*. **9**, 2273-2277 (2016).

13. Koketsu, T. et al. Reversible magnesium and aluminium ions insertion in cation-deficient anatase TiO_2. *Nat. Mater*. **16**, 1142-1148 (2017).

14. Chen, C., Wang, J., Zhao, Q., Wang, Y. & Chen, J. Layered $Na_2Ti_3O_7$/ $MgNaTi_3O_7$/$Mg_{0.5}NaTi_3O_7$ nanoribbons as high-performance anode of rechargeable Mg-ion batteries. *ACS Energy Lett.* **1**, 1165-1172 (2016).
15. Zhou, L. et al. Interlayer-spacing-regulated $VOPO_4$ nanosheets with fast kinetics for high-capacity and durable rechargeable magnesium batteries. *Adv. Mater.* **30**, 1801984 (2018).
16. Yoo, H. D. et al. Fast kinetics of magnesium monochloride cations in interlayer-expanded titanium disulfide for magnesium rechargeable batteries. *Nat. Commun.* **8**, 339 (2017).
17. Xue, X. et al. One-step synthesis of 2-ethylhexylamine pillared vanadium disulfide nanoflowers with ultralarge interlayer spacing for high-performance magnesium storage. *Adv. Energy Mater.* **9**, 1900145 (2019).
18. Pei, C. et al. Interchain-expanded vanadium tetrasulfide with fast kinetics for rechargeable magnesium batteries. *ACS Appl. Mater. Interfaces* **11**, 31954-31961 (2019).
19. Liang, Y. et al. Interlayer-expanded molybdenum disulfide nanocomposites for electrochemical magnesium storage. *Nano Lett.* **15**, 2194-2202 (2015).
20. Tang, H. et al. Alkali ions pre-intercalated layered vanadium oxide nanowires for stable magnesium ions storage. *Nano Energy* **58**, 347-354 (2019).
21. Wang, Y. et al. Atomic substitution enabled synthesis of vacancy-rich two-dimensional black TiO_{2-x} nanoflakes for high-performance rechargeable magnesium batteries. *ACS Nano* **12**, 12492-12502 (2018).
22. Gu, Y., Katsura, Y., Yoshino, T., Takagi, H. & Taniguchi, K. Rechargeable magnesium-ion battery based on a $TiSe_2$-cathode with *d-p* orbital hybridized electronic structure. *Sci. Rep.* **5**, 12486 (2015).
23. Yoo, H. D. et al. Intercalation of magnesium into a layered vanadium oxide with high capacity. *ACS Energy Lett.* **4**, 1528-1534 (2019).
24. Andrews, J. L. et al. Reversible Mg-ion insertion in a metastable one-dimensional polymorph of V_2O_5. *Chem.* **4**, 564-585 (2018).
25. Xue, X. et al. Electrochemical Mg^{2+} displacement driven reversible copper extrusion/intrusion reactions for high-rate rechargeable magnesium batteries. *Adv. Funct. Mater.* **31**, 2009394 (2021).
26. Xiong, F. et al. Magnesium storage performance and mechanism of CuS cathode. *Nano Energy* **47**, 210-216 (2018).

27. Hasegawa, G. et al. Reversible electrochemical insertion/extraction of magnesium ion into/from robust NASICON-type crystal lattice in a $Mg(BF_4)_2$-based electrolyte. *ACS Appl. Energy Mater*. **3**, 6824-6833 (2020).
